# Supplementary material for: Genomic characterization of cervical lymph node metastases in papillary thyroid carcinoma following the Chornobyl accident
Source: Nat Commun. 2024 Jun 13;15:5053. doi: 10.1038/s41467-024-49292-z (PMC11176192; doi:10.1038/s41467-024-49292-z)
Supplement: Supplementary file 1 — Supplementary Information [file 41467_2024_49292_MOESM1_ESM.pdf]

Supplementary Figure S1. Distribution of available pathologic characteristics (pathologic T classification, multifocality, and primary lesion size) among the N=440 PTC tumors in our primary study population (A), N=68 PTC tumors from non-overlapping individuals from two previous Chornobyl studies (B), and N=326 PTC tumors from TCGA (C). Percentages >20% are shown. Source data are provided as follows: Table S2 provides all counts and percentages.
Abbreviations: cervical lymph node metastases (cLNM), yes/no (N1/N0); distant metastases, yes/no (M1/M0), papillary thyroid carcinoma (PTC), The Cancer Genome Atlas (TCGA).

A

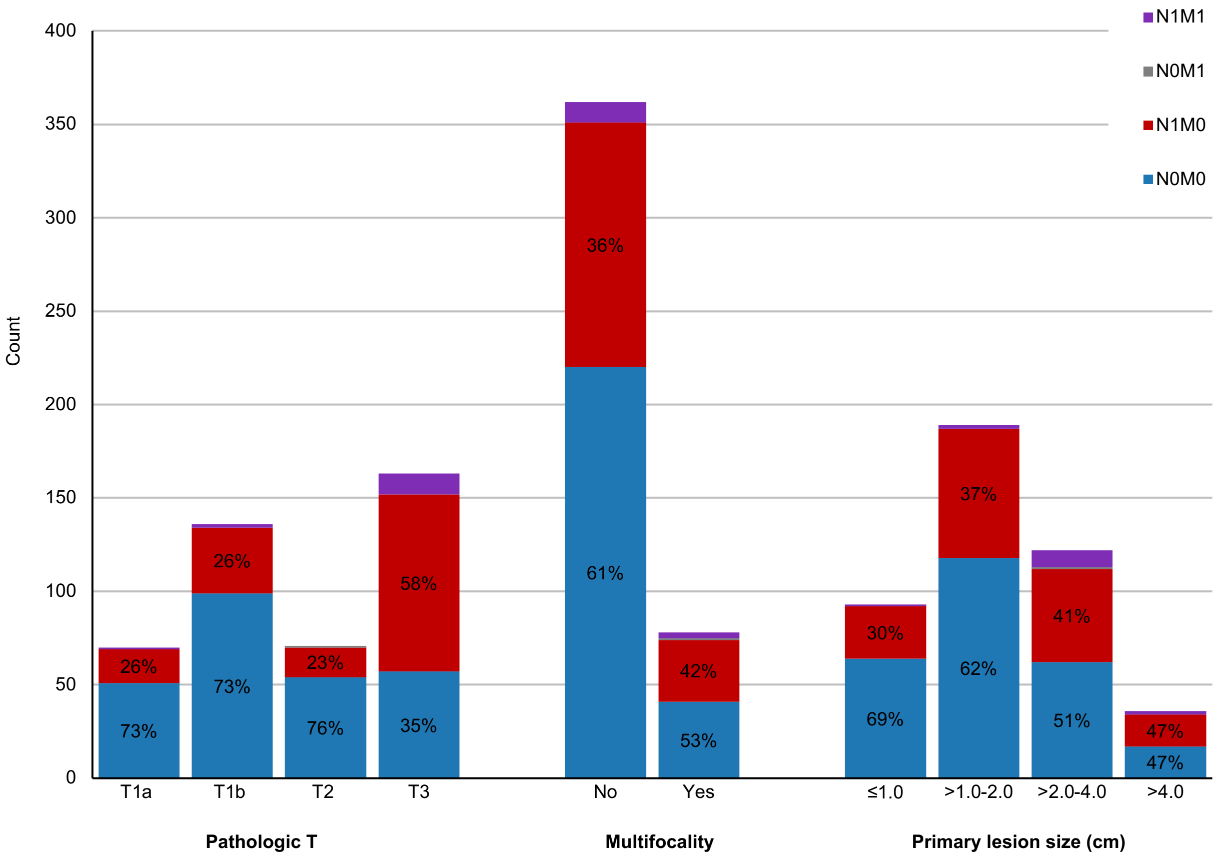

B

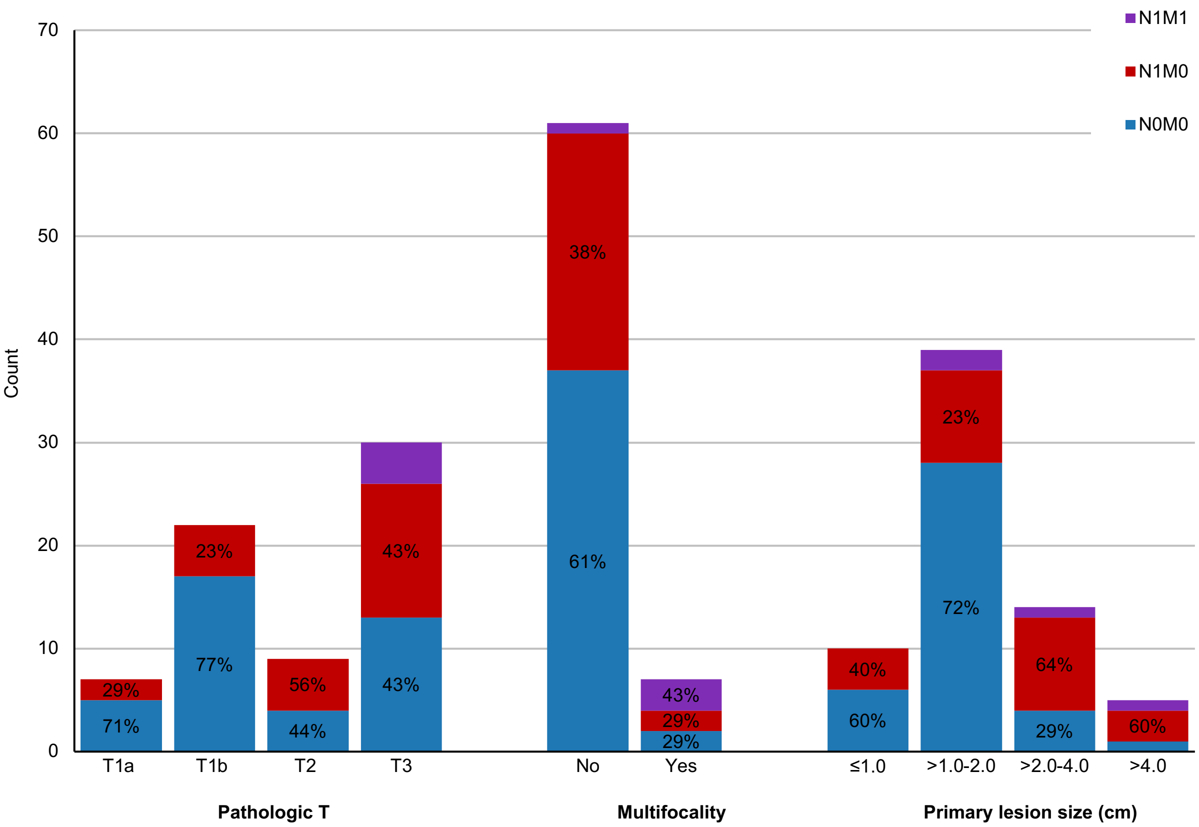
N1M1
N1M0
N0M0
70
60
50
40
30
20
10
0
Count
29%
71%
23%
77%
56%
44%
43%
43%
38%
61%
43%
29%
29%
40%
60%
23%
72%
64%
29%
60%
T1a
T1b
T2
T3
No
Yes
≤1.0
>1.0-2.0
>2.0-4.0
>4.0
Pathologic T
Multifocality
Primary lesion size (cm)

C

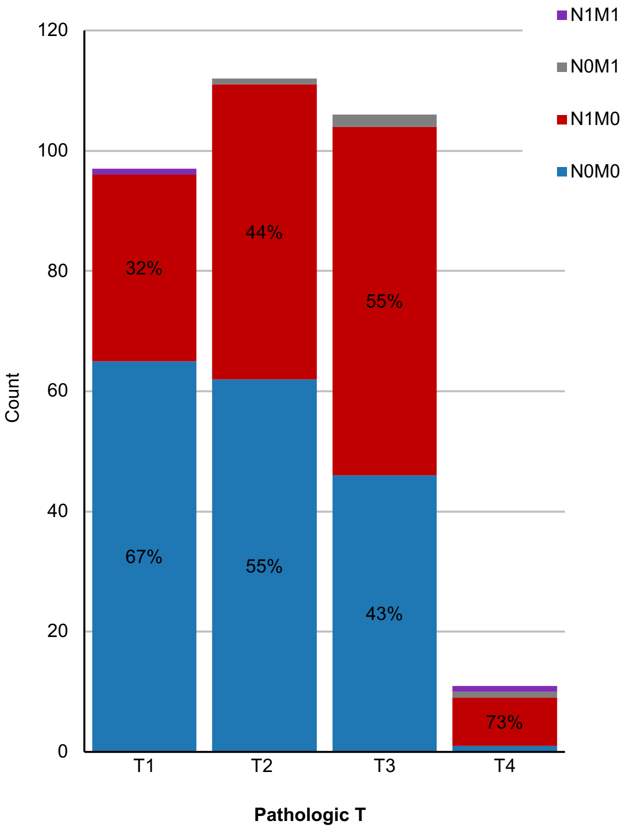
N1M1
N0M1
N1M0
N0M0
120
100
80
60
40
20
0
Count
32%
67%
44%
55%
55%
43%
73%
T1
T2
T3
T4
Pathologic T

Supplementary Figure S2. Distribution of driver, age at PTC*, and presence of cervical lymph node metastases (cLNM) at diagnosis among the N=428 PTC tumors in our primary study population with a final designated driver. Percentages >20% are shown. Source data are provided as follows: Further breakdowns by age are provided in Table S6.

Abbreviations: cervical lymph node metastases (cLNM), papillary thyroid carcinoma (PTC).

*Analyses were stratified at age 30 years, reflecting the mean age at PTC diagnosis among exposed individuals.

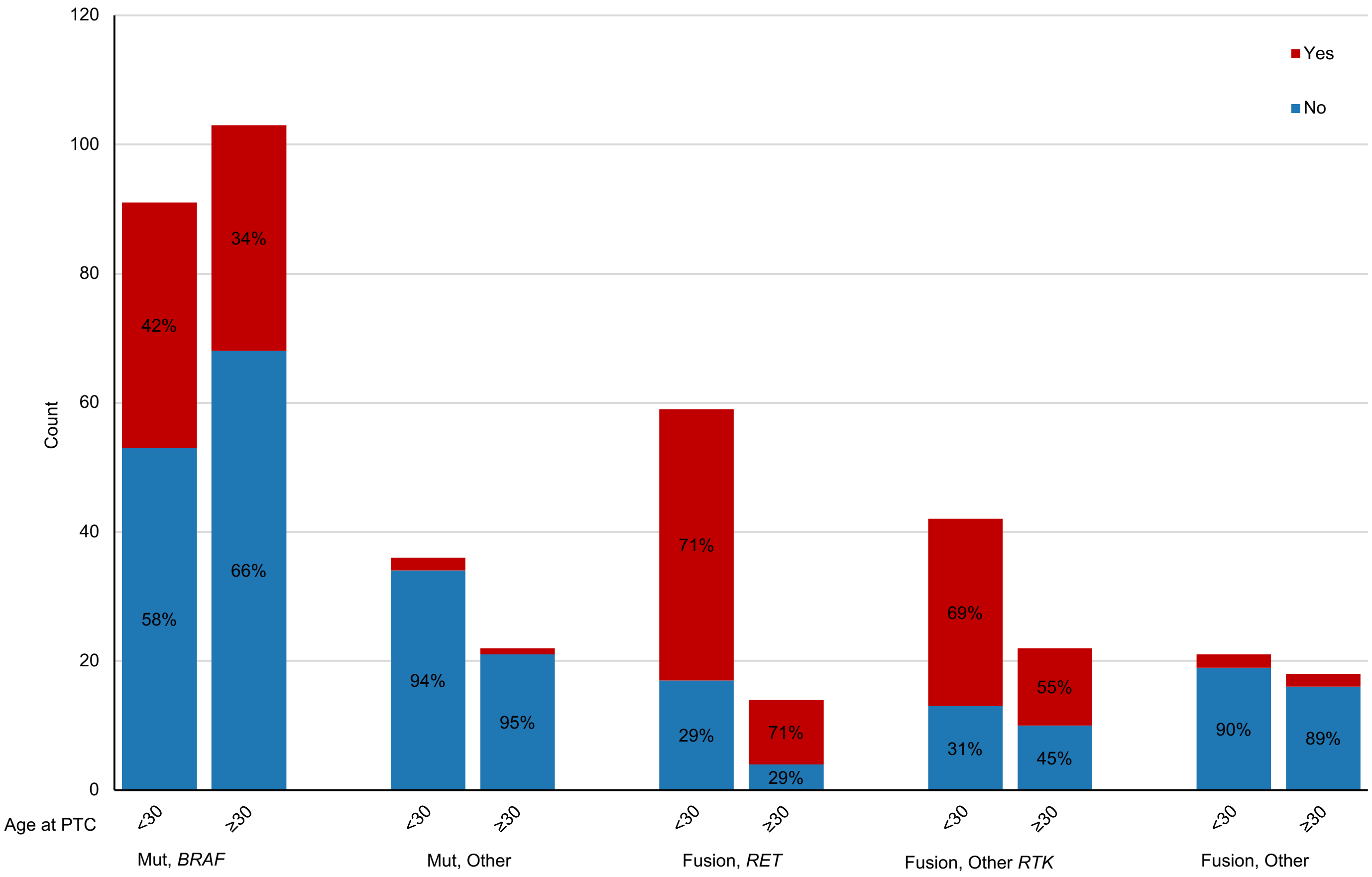

Supplementary Figure S3. Distribution of cLNM at diagnosis among the N=68 non-overlapping individuals from two previous Chornobyl studies (A) and the N=326 individuals from TCGA (B), by driver type and gene. Percentages >20% are shown. $P_{heterogeneity}$ represents a two-sided P-value calculated using likelihood ratio tests, comparing model fit with and without the variable of interest. Source data are provided as follows: Table S4 provides all counts and percentages, including information on the other mutations and fusions.

Abbreviations: cervical lymph node metastases (cLNM), The Cancer Genome Atlas (TCGA).

A

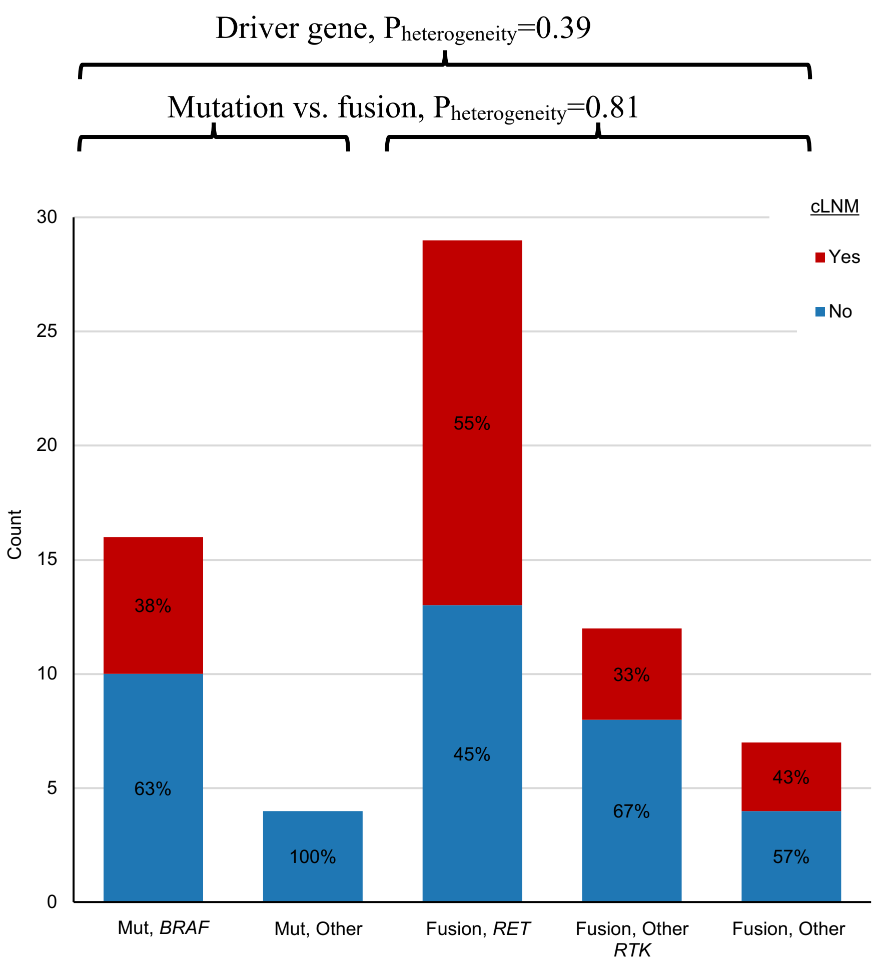

B

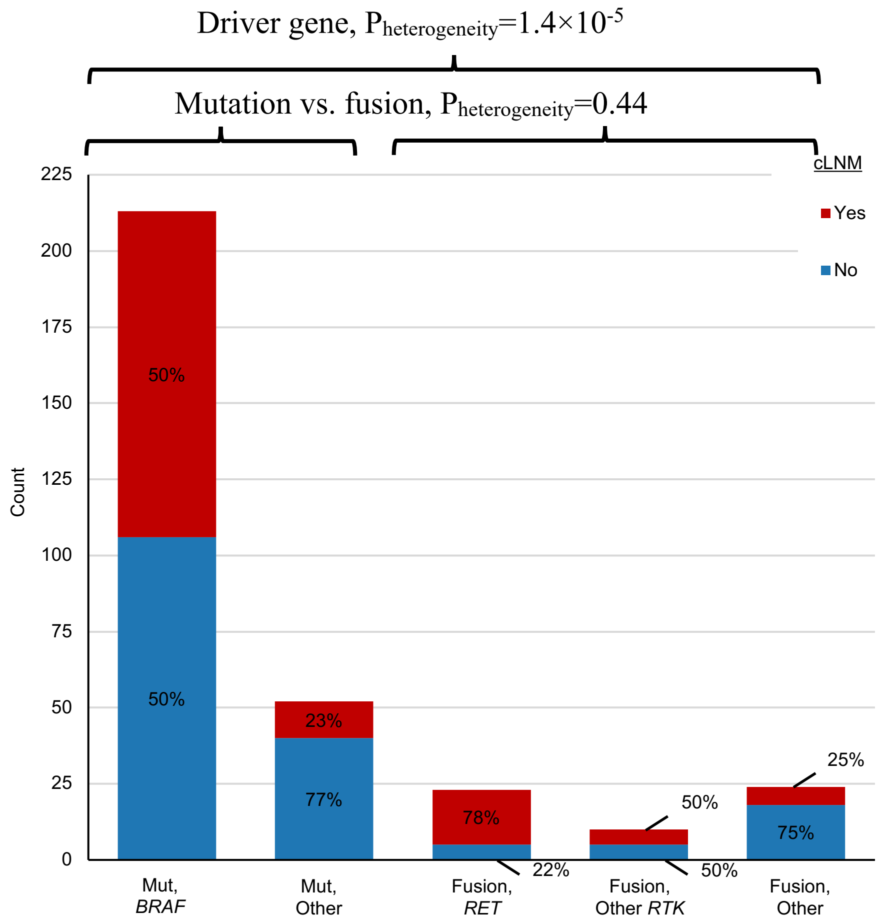

Driver gene, $P_{heterogeneity}=1.4\times10^{-5}$
Mutation vs. fusion, $P_{heterogeneity}=0.44$
cLNM
Yes
No
Count
225
200
175
150
125
100
75
50
25
0
50%
50%
23%
77%
78%
22%
50%
50%
25%
75%
Mut, *BRAF*
Mut, Other
Fusion, *RET*
Fusion, Other *RTK*
Fusion, Other

Supplementary Figure S4. Distribution of WGS, mRNA-seq, miRNA-seq, DNA methylation profiling, and relative telomere length quantification for N=47 individuals with cLNM samples, overall (A) and restricted to high purity samples* (B).

Sample type abbreviations: cervical lymph node metastases (LNM), normal blood (NB), non-tumor thyroid tissue (NT), primary tumor tissue (PT).

*High purity=tumor cell purity >20%.

A

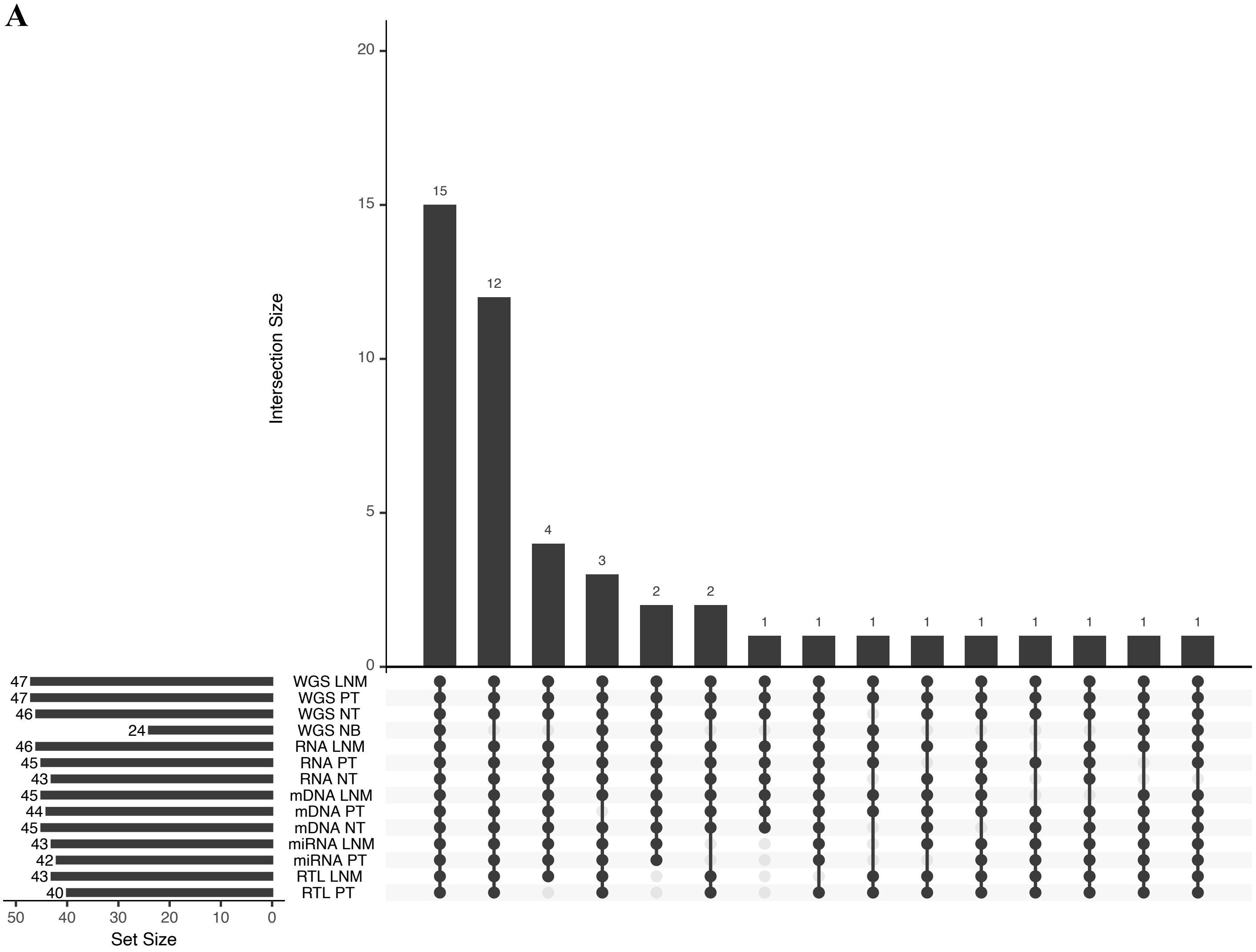

B

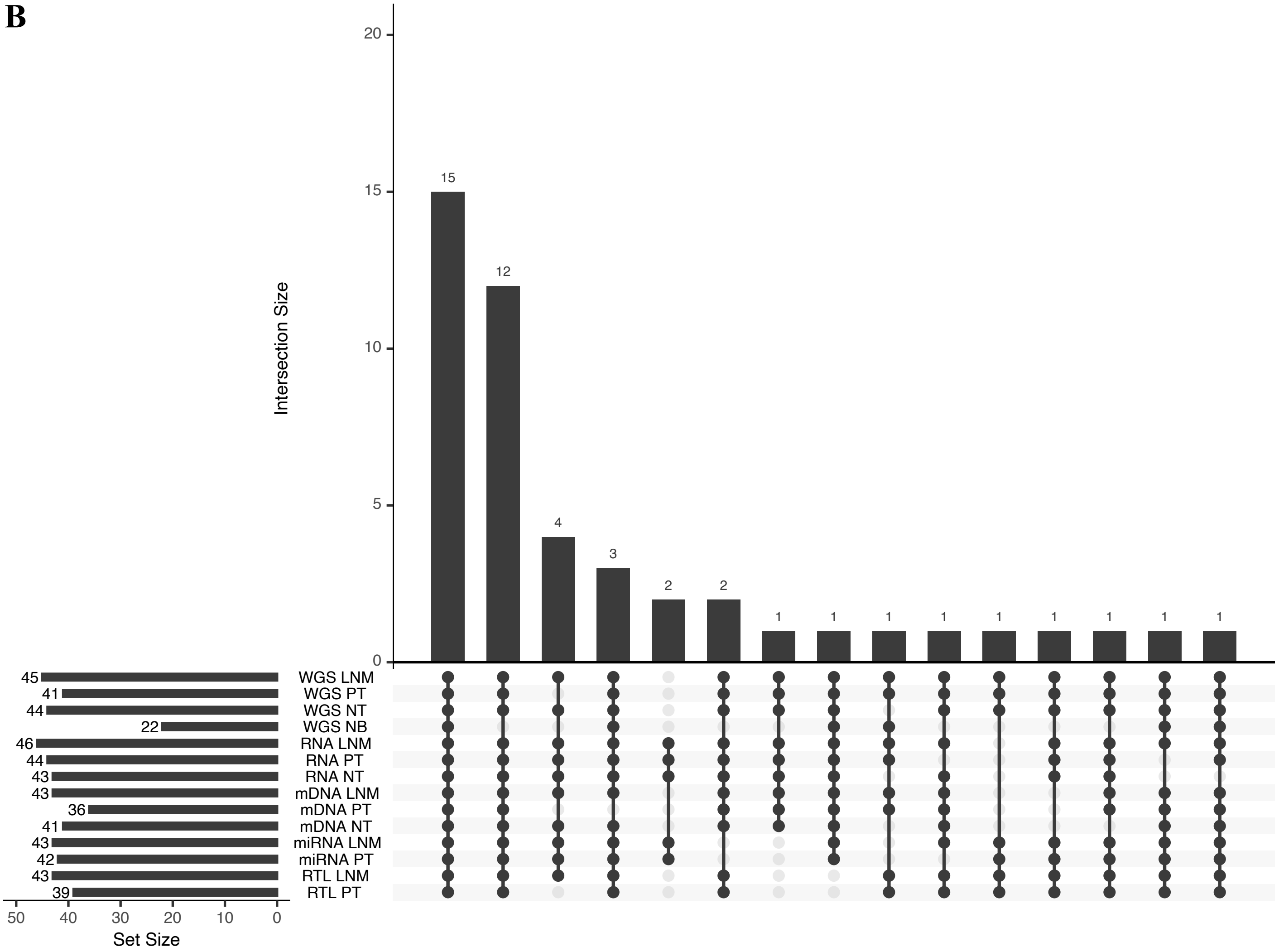
Intersection Size
20
15
10
5
0
15
12
4
3
2
2
1
1
1
1
1
1
1
1
1
WGS LNM
WGS PT
WGS NT
WGS NB
RNA LNM
RNA PT
RNA NT
mDNA LNM
mDNA PT
mDNA NT
miRNA LNM
miRNA PT
RTL LNM
RTL PT
45
41
44
22
46
44
43
43
36
41
43
42
43
39
50
40
30
20
10
0
Set Size

Supplementary Figure S5. Scatter plots of the distribution of SNVs (A), small deletions (B), and small insertions (C) in N=41 paired cLNM-PT samples. Source data are provided as follows: Table S11 provides counts. Figure 3 provides distributions in cLNM, paired PT, and matched PT samples.
Abbreviations: cervical lymph node metastases (cLNM), primary tumor (PT), single nucleotide variant (SNV).

A

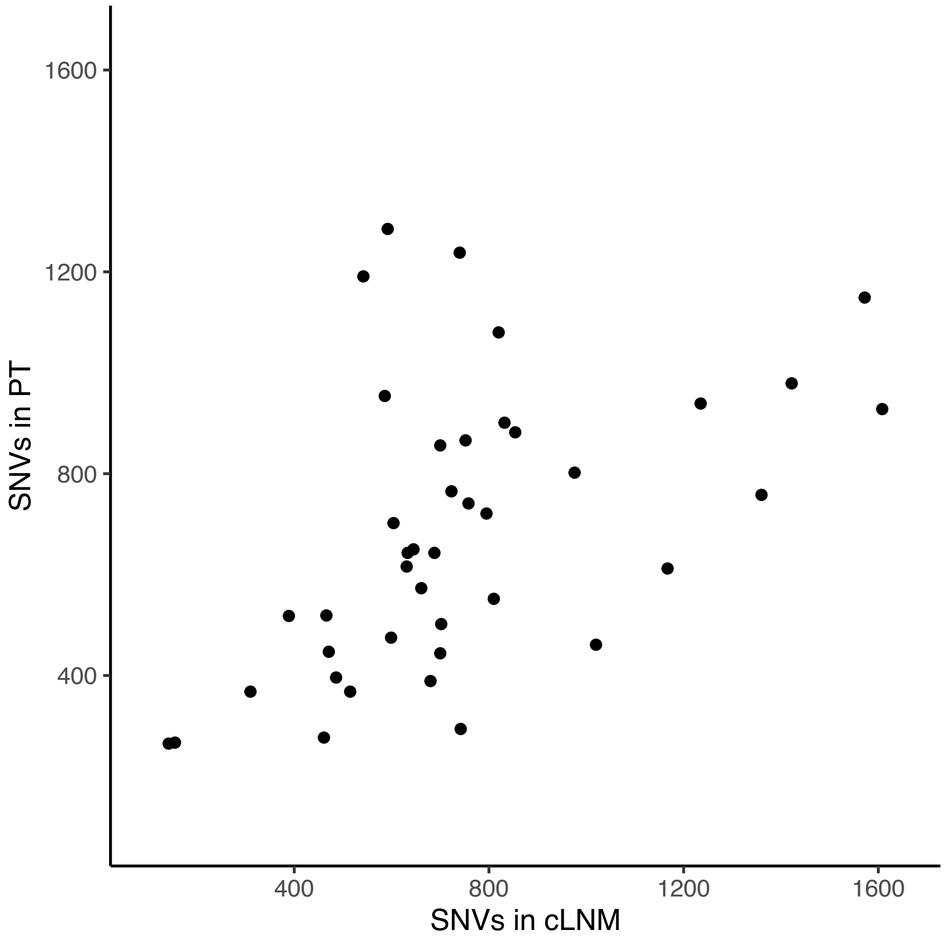

B

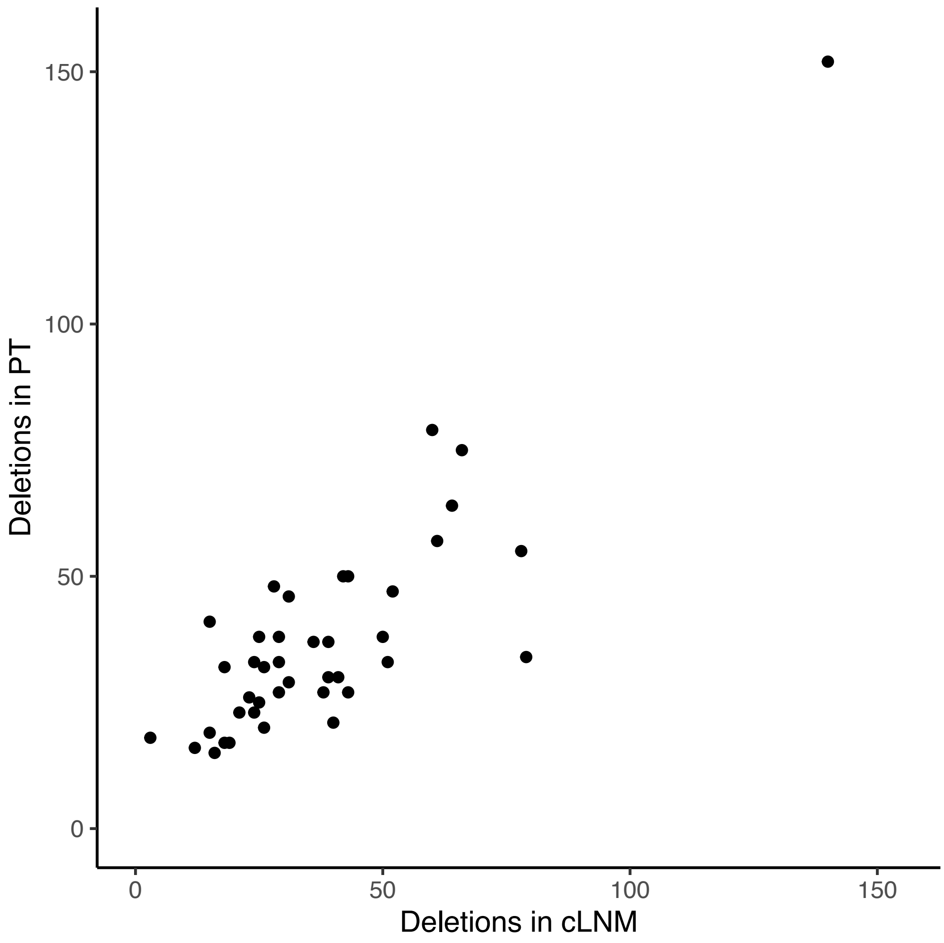

C

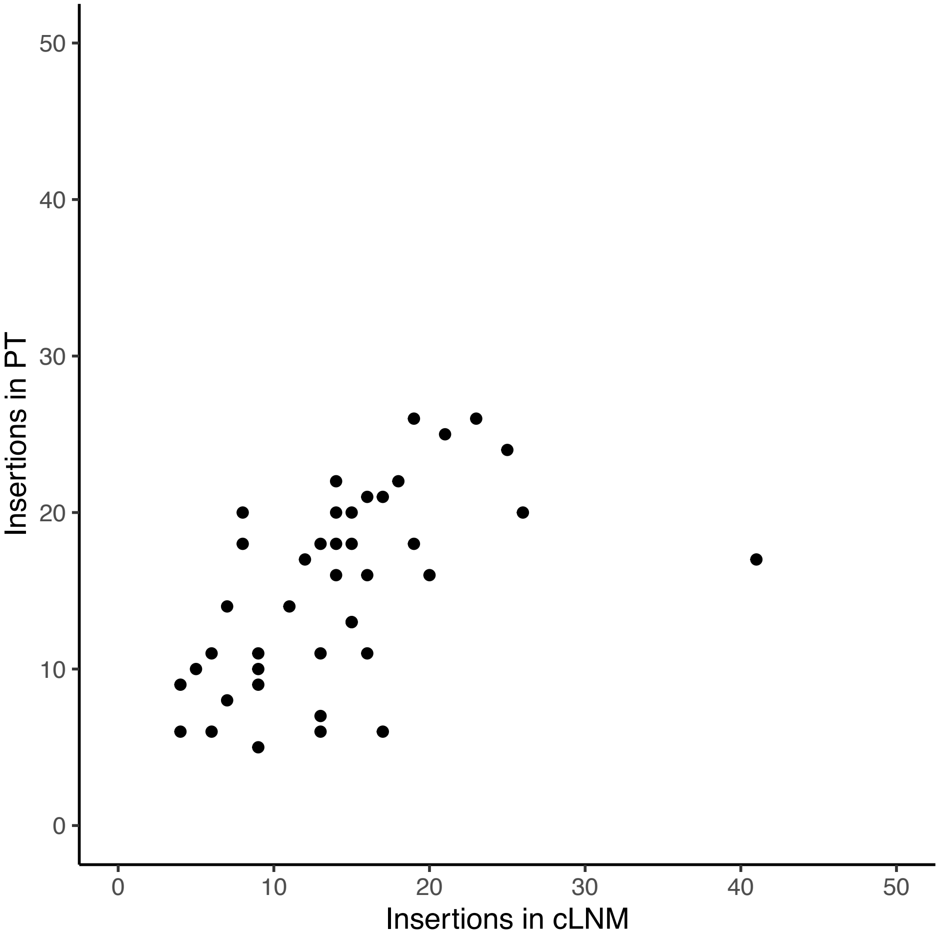

Insertions in PT
Insertions in cLNM
0
10
20
30
40
50
0
10
20
30
40
50

Supplementary Figure S6. Violin plots of the SNV distribution in N=41 paired cLNM-PT samples by shared versus private to the cLNM or PT samples, overall (A) and separately for clonal* (B) and subclonal (C) mutations. All box plots include the center line at the median, the box denotes the interquartile range (IQR), whiskers denote the rest of the data distribution, and outliers are denoted by points greater than ±1.5 × IQR. Source data are provided as follows: Table S11 provides counts.
* Clonal=cancer cell fraction ≥0.6, subclonal=cancer cell fraction<0.6.
Abbreviations: cervical lymph node metastases (cLNM), primary tumor (PT), single nucleotide variant (SNV).

A

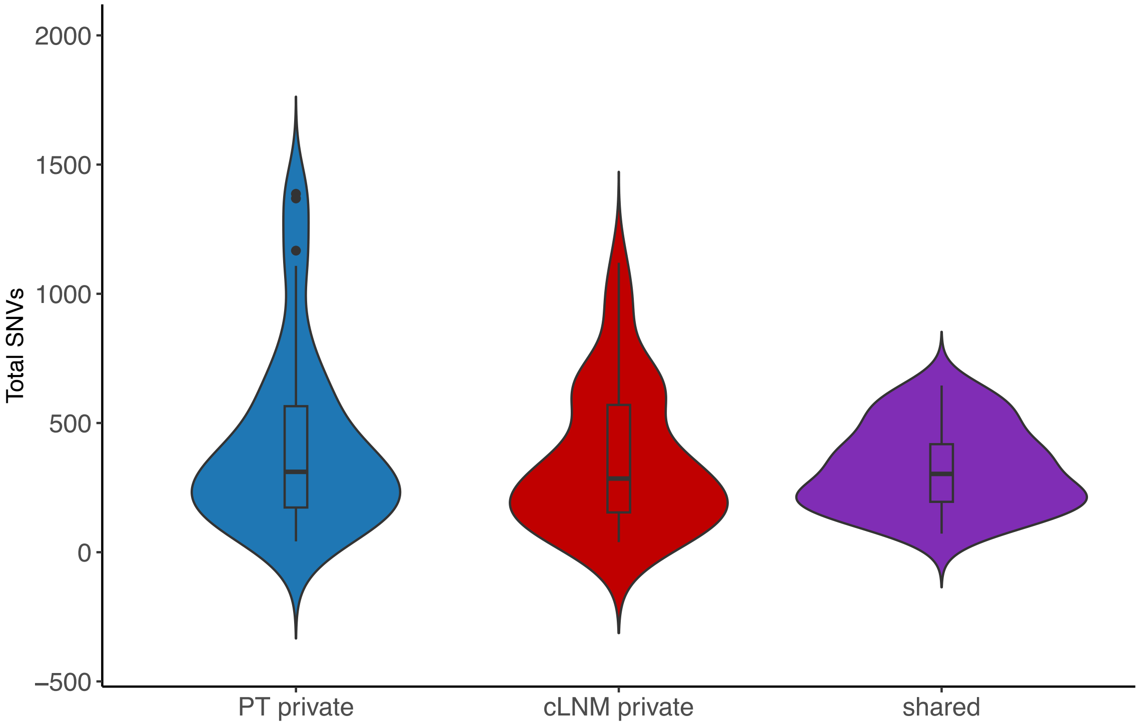

B

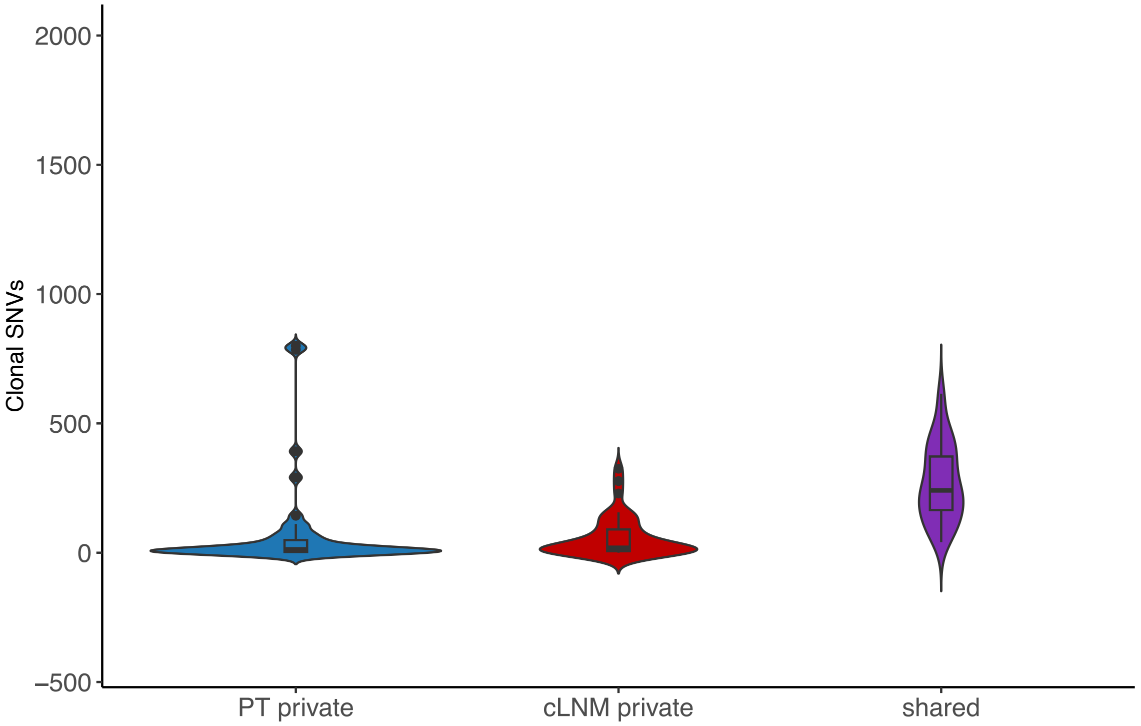
2000
1500
1000
500
0
−500
Clonal SNVs
PT private
cLNM private
shared

C

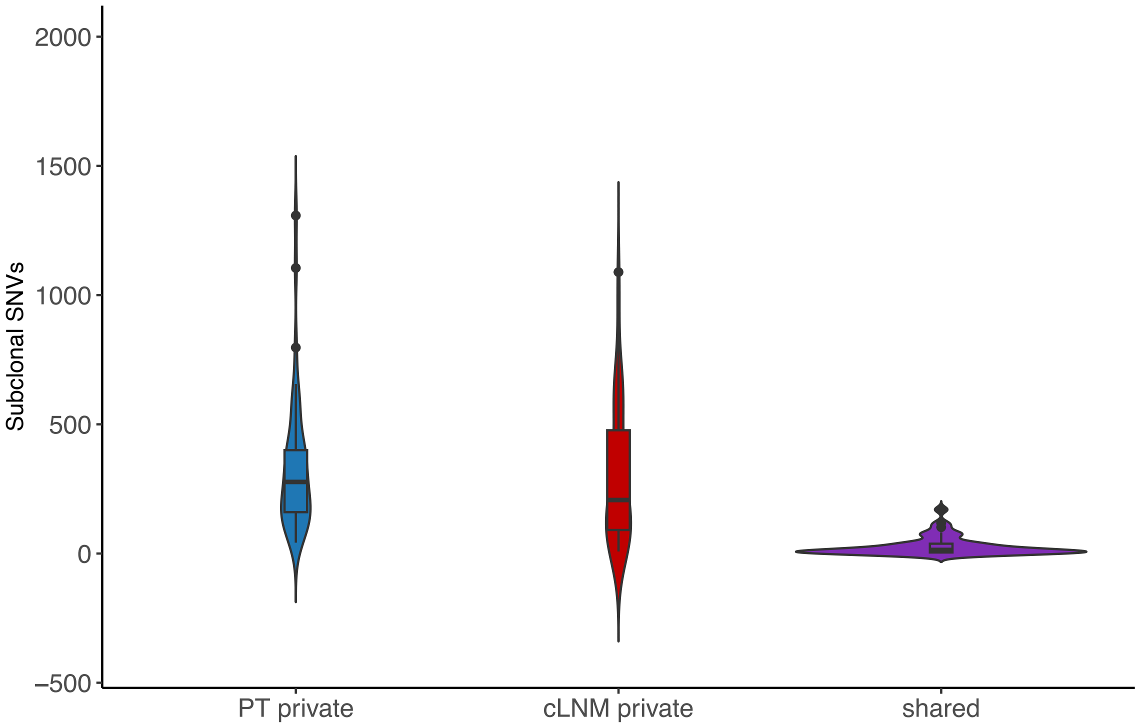
2000
1500
1000
500
0
−500
Subclonal SNVs
PT private
cLNM private
shared

Figure S7. Distribution of the SNVs in 41 paired cLNM-PT samples by clonality* and shared versus private to the cLNM or PT samples (A). Examples of samples with evidence for polyclonal seeding (B) or sampling bias (C).
Abbreviations: cervical lymph node metastases (cLNM), primary tumor (PT), single nucleotide variant (SNV).
* Clonal=cancer cell fraction≥0.6.

A

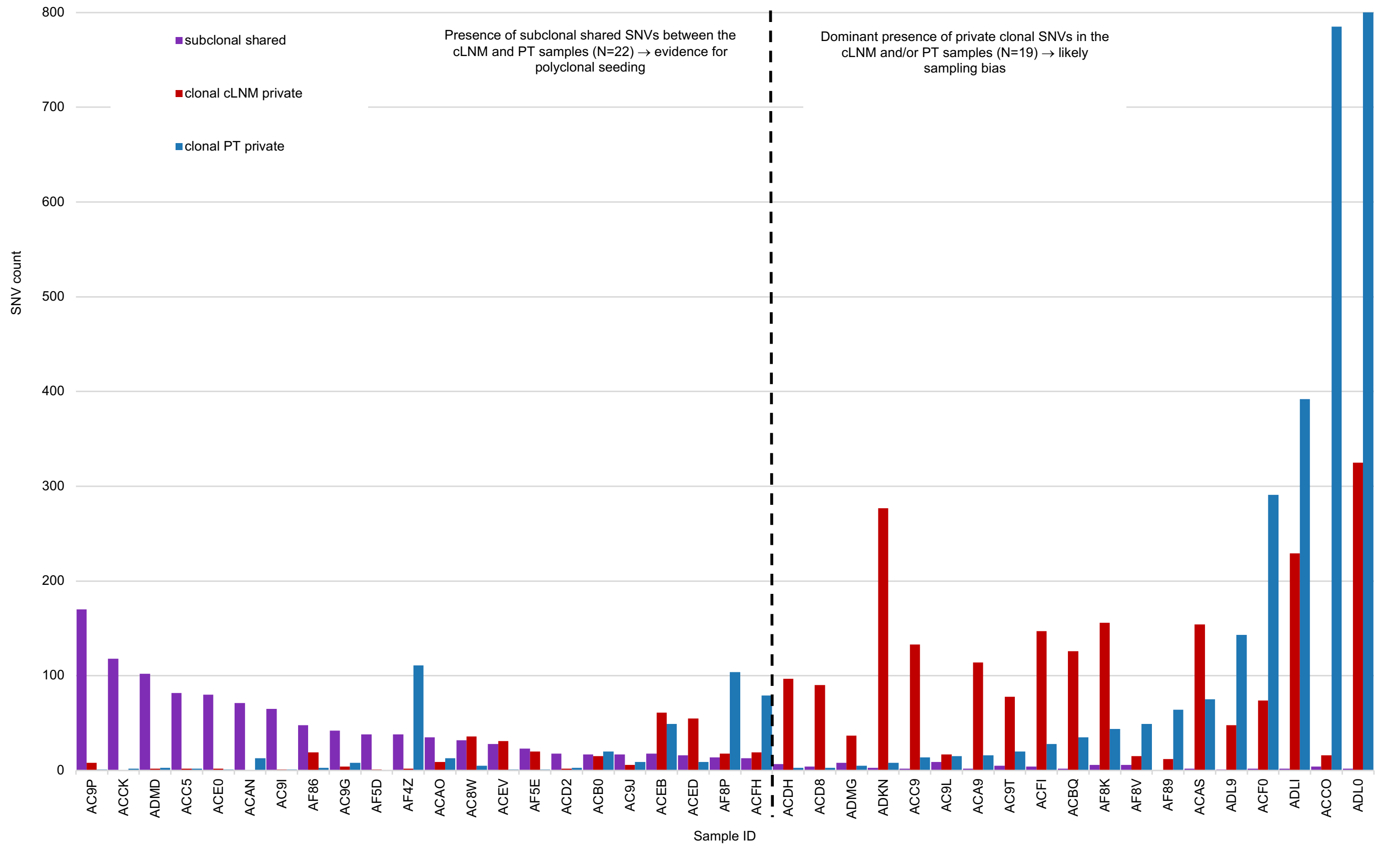

B

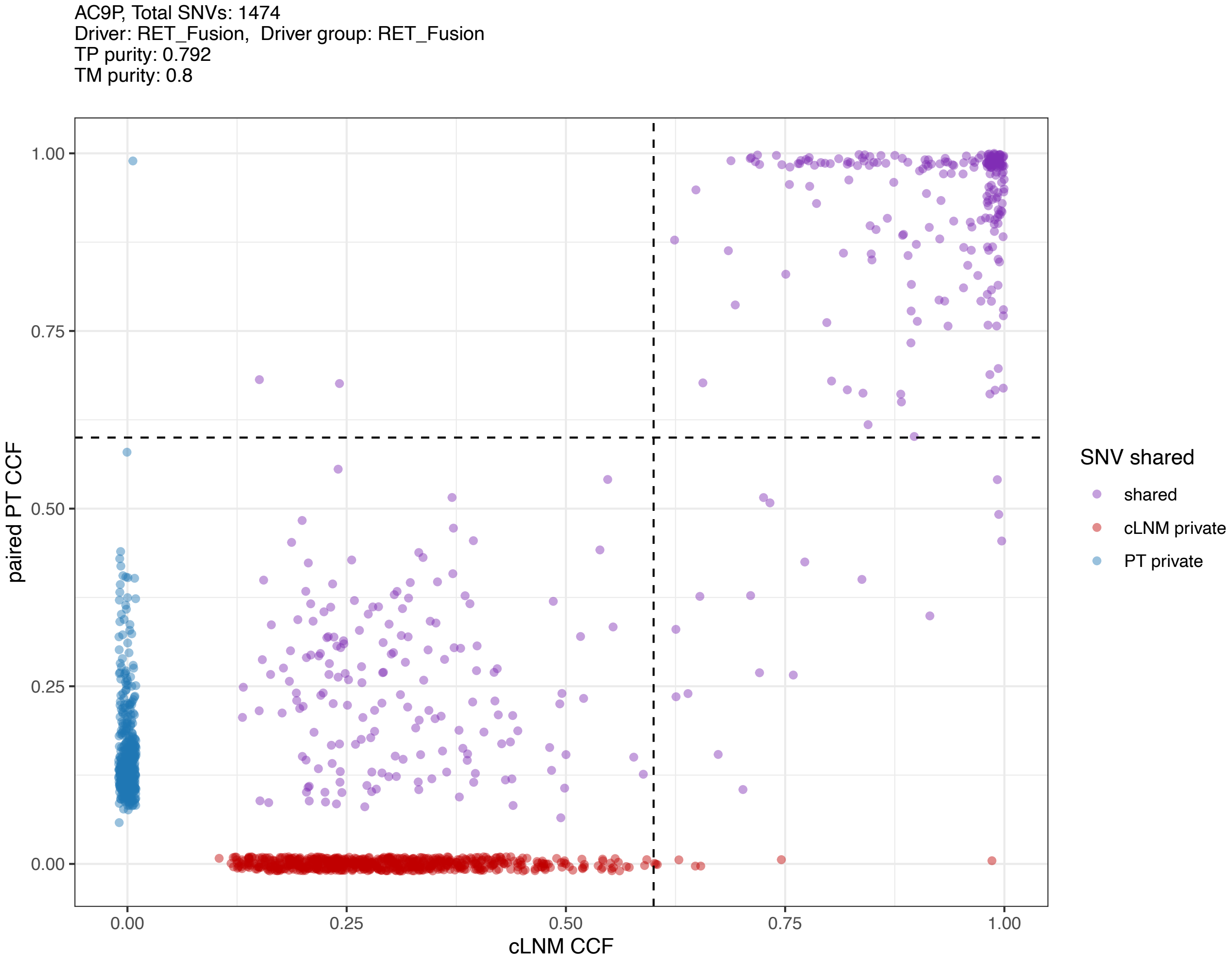
AC9P, Total SNVs: 1474
Driver: RET_Fusion, Driver group: RET_Fusion
TP purity: 0.792
TM purity: 0.8
paired PT CCF
cLNM CCF
0.00
0.25
0.50
0.75
1.00
SNV shared
shared
cLNM private
PT private

C

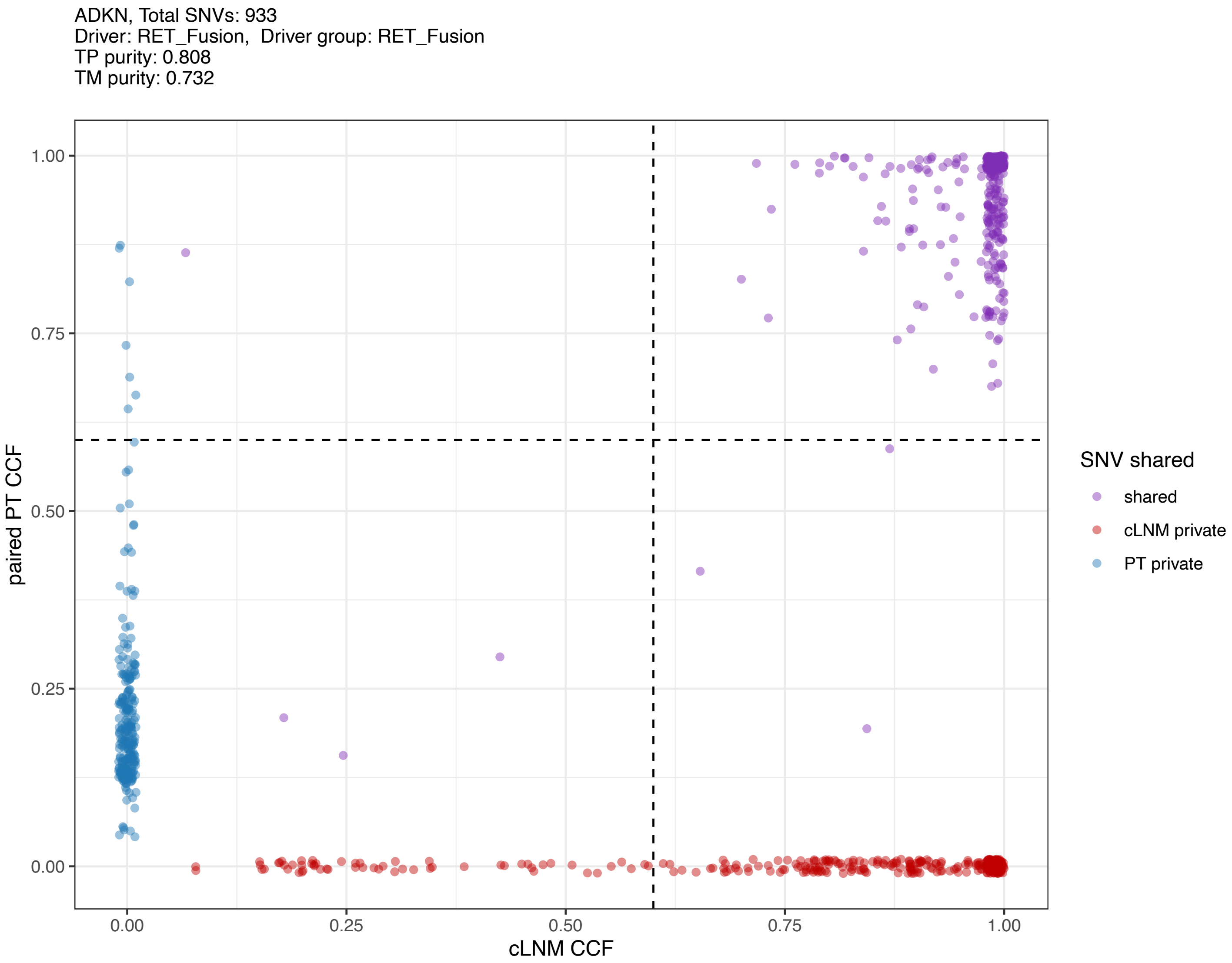
ADKN, Total SNVs: 933
Driver: RET_Fusion, Driver group: RET_Fusion
TP purity: 0.808
TM purity: 0.732
paired PT CCF
1.00
0.75
0.50
0.25
0.00
0.00
0.25
0.50
0.75
1.00
cLNM CCF
SNV shared
shared
cLNM private
PT private

Supplementary Figure S8. The distribution of principal component 1 (PC1) and 2 (PC2) from unsupervised hierarchical clustering analyses of mRNA sequencing data among cLNM, PT, and non-tumor thyroid tissue.

Abbreviations: cervical lymph node metastases (cLNM), non-tumor thyroid tissue (NT), primary tumor (PT).

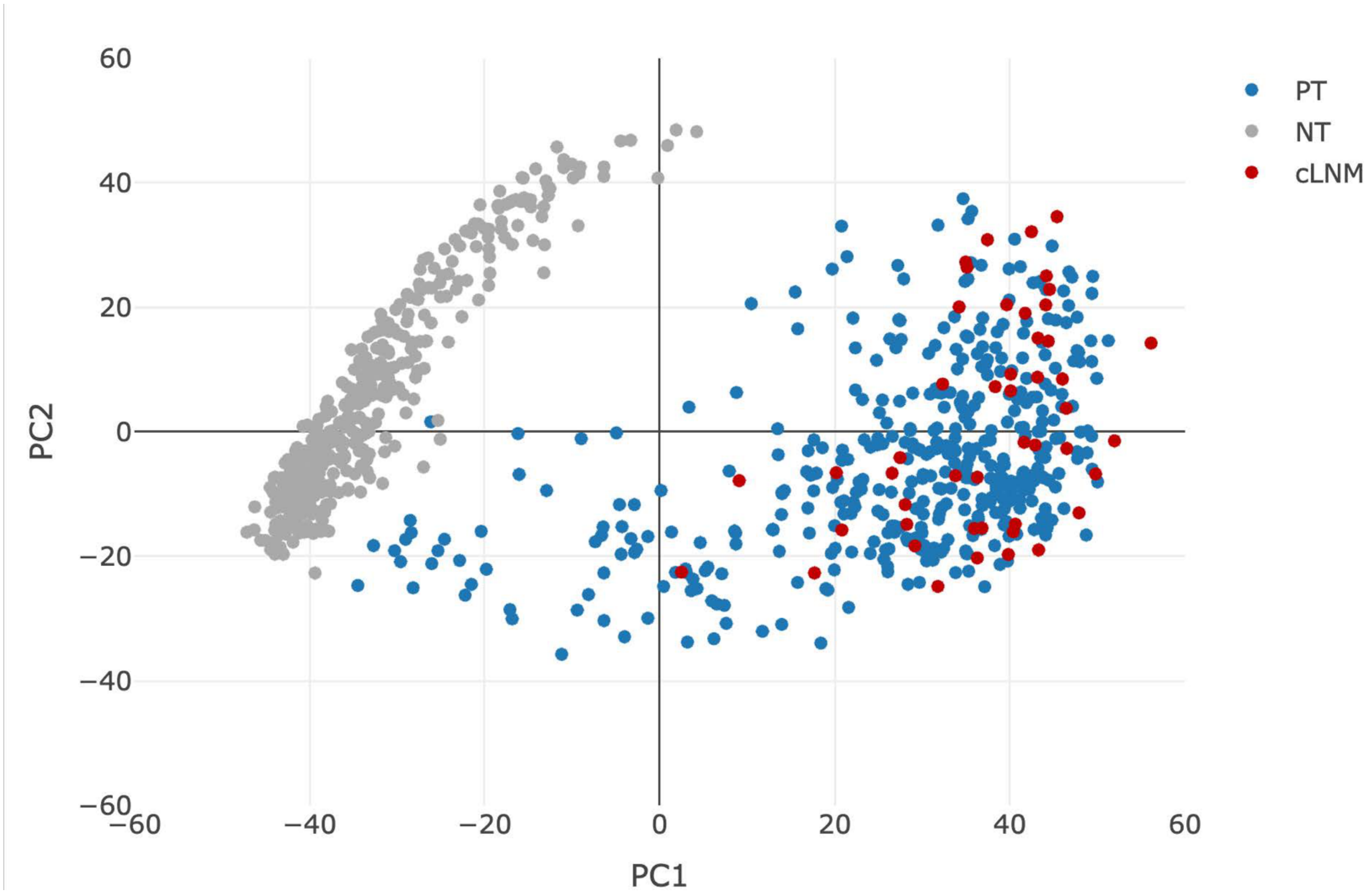

Supplementary Figure S9. Expression levels of *HOXC10* (A) and miR-196A2 (B) in each sample type, restricted to pediatric cases (age <20 years at PTC diagnosis). All box plots include the center line at the median, the box denotes the interquartile range (IQR), whiskers denote the rest of the data distribution, and outliers are denoted by points greater than ±1.5 × IQR.
Abbreviations: cervical lymph node metastases (cLNM), non-tumor thyroid tissue (NT), primary tumor (PT).

A

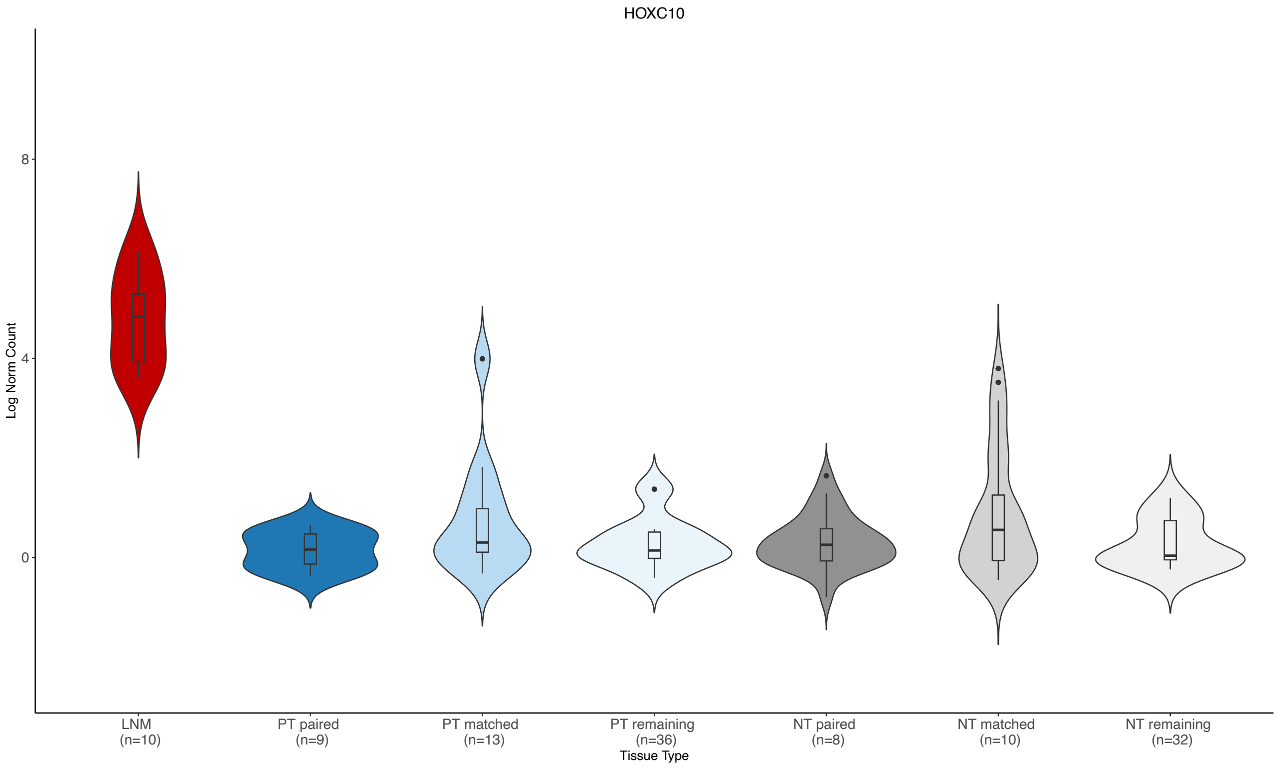

B

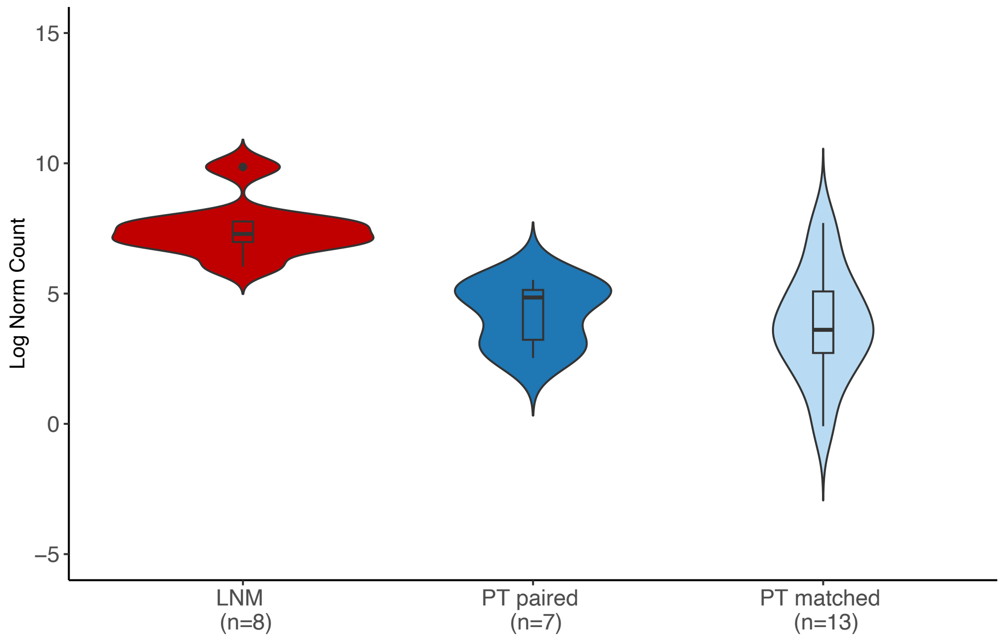

Supplementary Figure S10. Expression of *HOXC10* (A), *HOTAIR* (B), and *BRINP3* (C) in cell types with >5000 cells from the Tabula Sapiens Consortium single-cell mRNAseq data (*Science*, 2022).

A

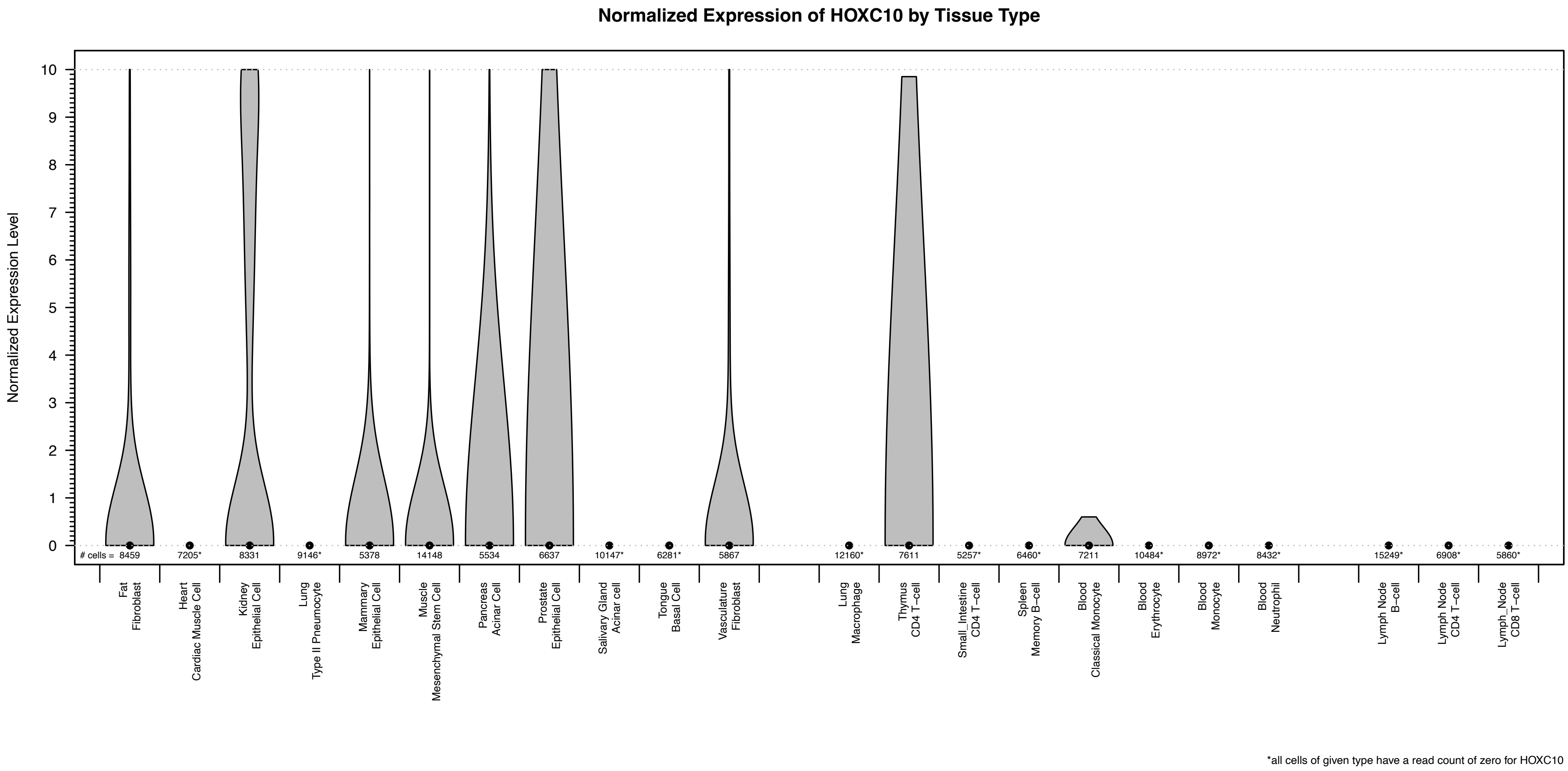

B

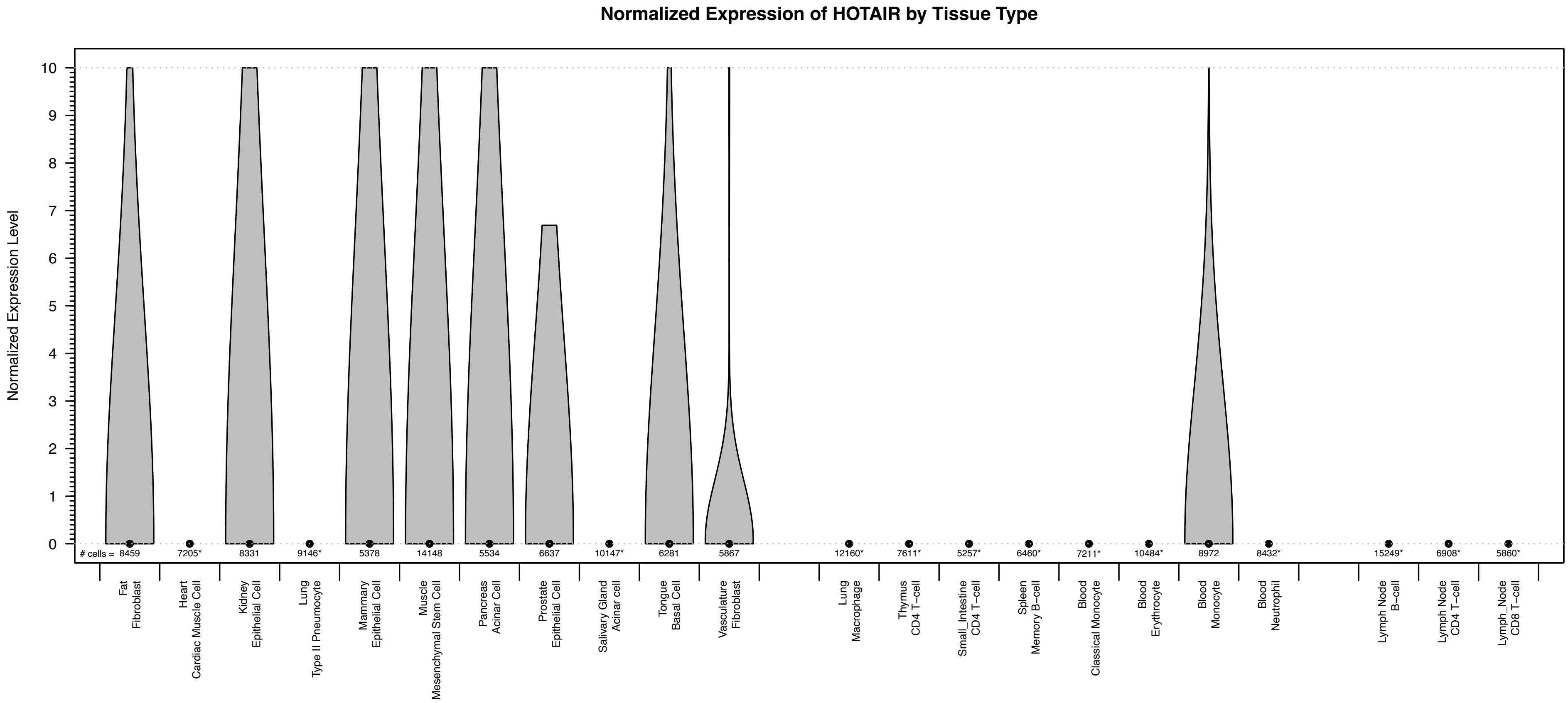

*all cells of given type have a read count of zero for HOTAIR

C

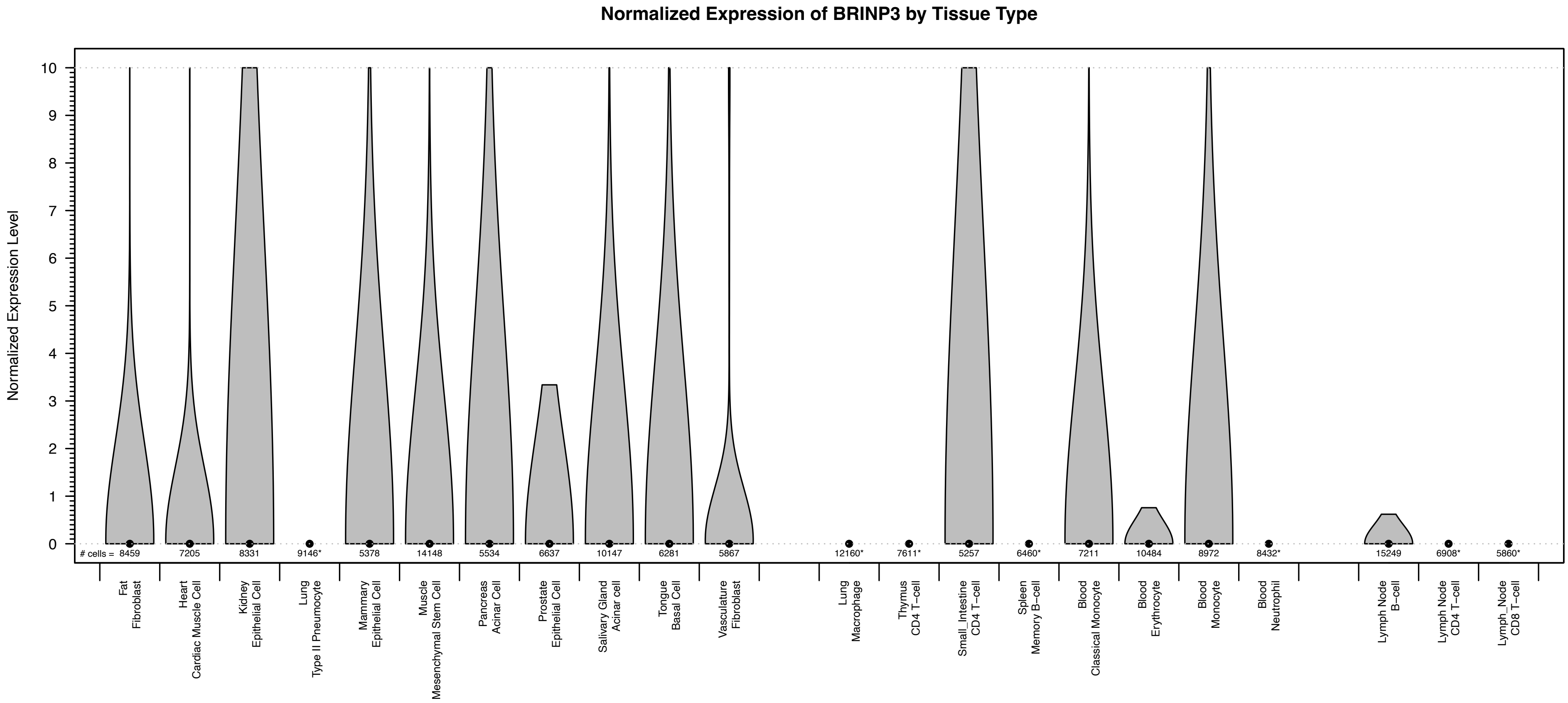

*all cells of given type have a read count of zero for BRINP3

Supplementary Figure S11. Composition of cell types in cLNM and PT samples estimated from mRNA-seq data using CIBERSORTx. All box plots include the center line at the median, the box denotes the interquartile range (IQR), whiskers denote the rest of the data distribution, and outliers are denoted by points greater than ±1.5 × IQR.
Abbreviations: cervical lymph node metastases (cLNM), primary tumor (PT).

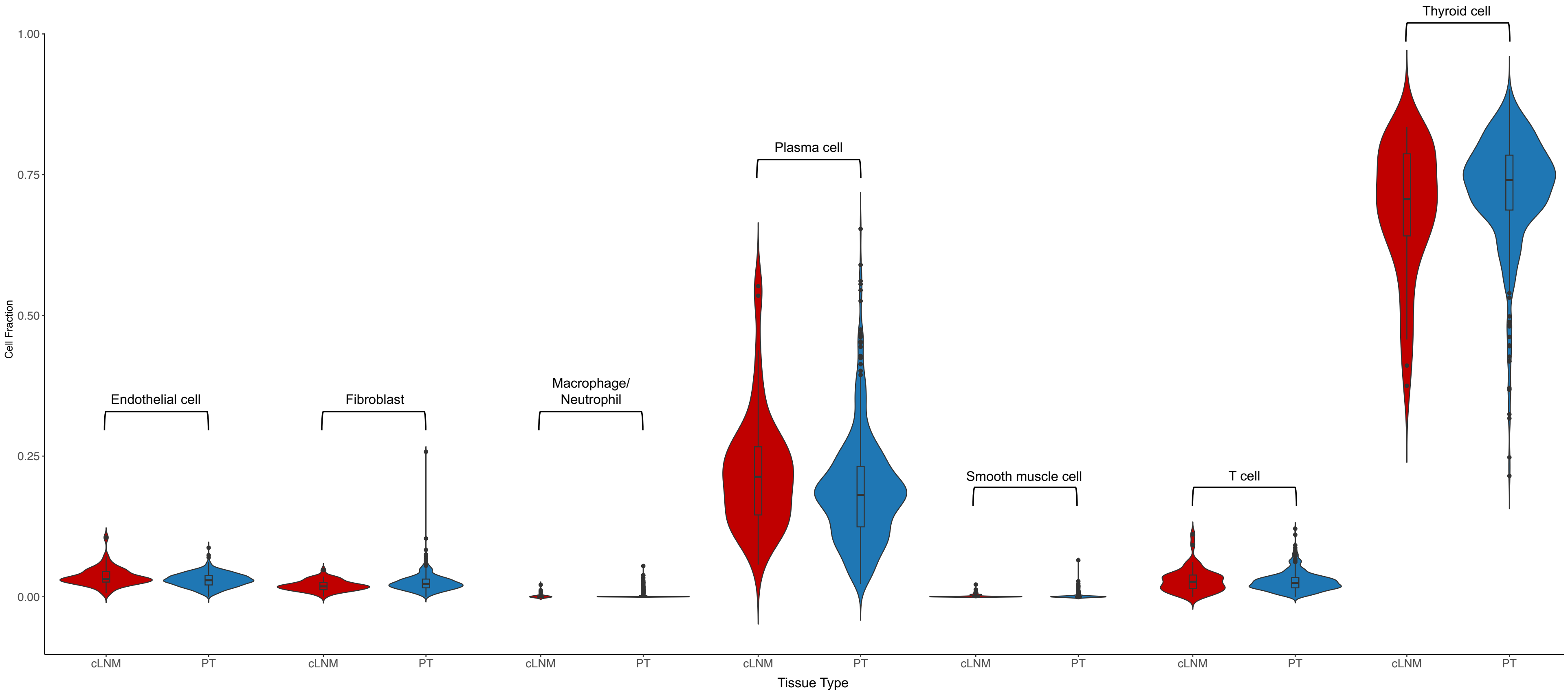

Supplementary Figure S12. Composition of immune cell types estimated from DNA methylation profiles in cLNM, paired PT, and matched PT samples. All box plots include the center line at the median, the box denotes the interquartile range (IQR), whiskers denote the rest of the data distribution, and outliers are denoted by points greater than ±1.5 × IQR.

Abbreviations: B cell: CD19+ B cell; CD4T: CD4+ T cell; CD8T: CD8+ T cell; cervical lymph node metastases (cLNM), NK: Natural killer cell, primary tumor (PT).

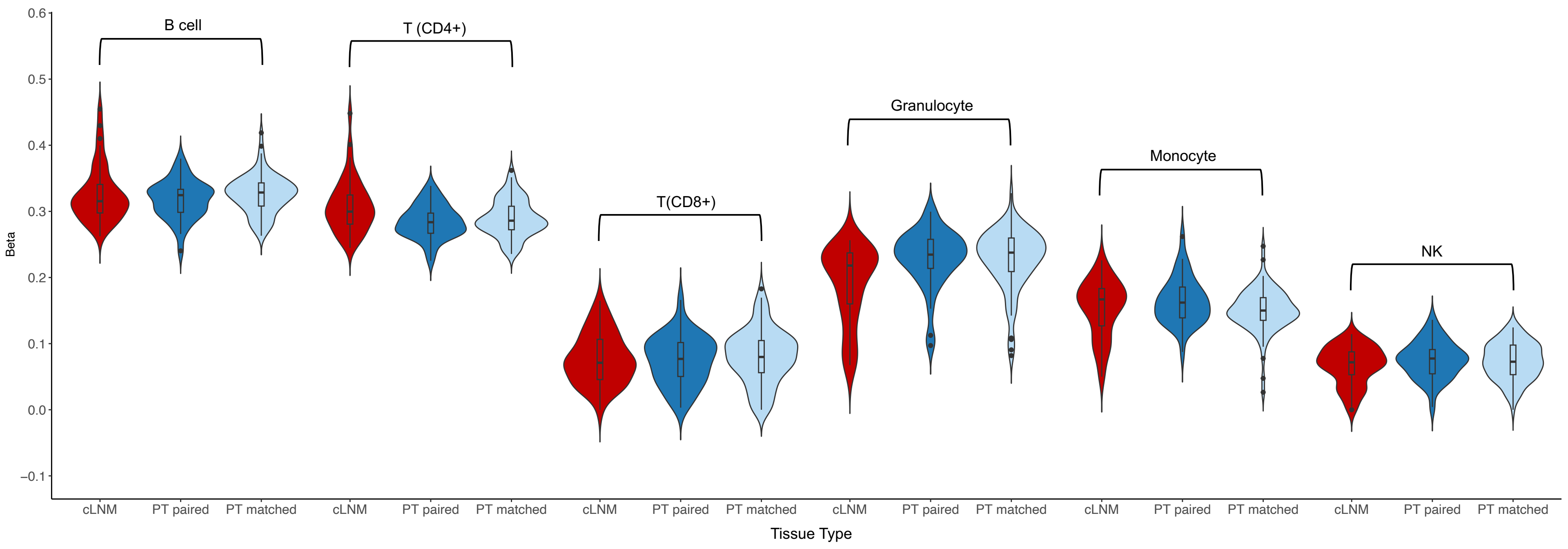

Supplementary Figure S13. eFORGE analysis for 68 probes with P_{adj}<0.05. Y-axis shows eFORGE enrichment (−log10 p-value) of the CpG set with DNase I hotspots for a range of tissue samples. X-axis indicates tissues/cell type samples used in the analysis. No samples were significantly enriched for immune cell types (see "Blood" below) or any other cell type or tissue in this analysis. Two-sided P-values were adjusted using the standard Benjamini & Hochberg false discovery rate (FDR) method.

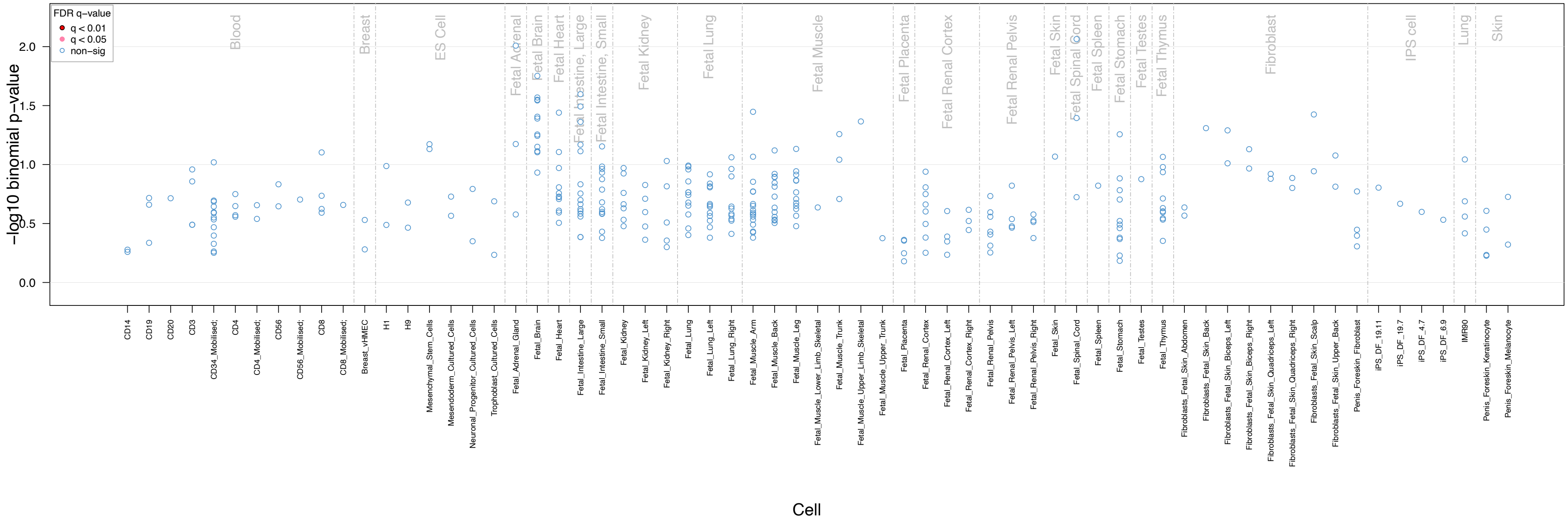

Supplementary Table S1. Demographic and clinical factors for patients with PTC included in our primary study population as well as previously published data from Chornobyl and TCGA.

Characteristic	Primary study population (N=440) N (%)	Other Chornobyl samples* (N=68) N (%)	TCGA† (N=326) N (%)
Sex			
Female	335 (76.1)	46 (67.6)	241 (73.9)
Male	105 (23.9)	22 (32.4)	85 (26.1)
Age at PTC (years)			
<15	15 (3.4)	9 (13.2)	0 (0.0)
15-19	50 (11.4)	23 (33.8)	5 (1.5)
20-24	74 (16.8)	14 (20.6)	18 (5.5)
25-29	117 (26.6)	14 (20.6)	20 (6.1)
30-34	125 (28.4)	8 (11.8)	43 (13.2)
35-39	43 (9.8)	0 (0.0)	41 (12.6)
40-44	16 (3.6)	0 (0.0)	33 (10.1)
45-49	0 (0.0)	0 (0.0)	39 (12.0)
50-54	0 (0.0)	0 (0.0)	36 (11.0)
55-59	0 (0.0)	0 (0.0)	24 (7.4)
60-64	0 (0.0)	0 (0.0)	21 (6.4)
65-69	0 (0.0)	0 (0.0)	16 (4.9)
70-74	0 (0.0)	0 (0.0)	15 (4.6)
≥75	0 (0.0)	0 (0.0)	15 (4.6)
Mean (± SD)	*28.0 (± 6.9)*	*21.5 (± 6.2)*	*46.0 (± 15.4)*
Radiation dose (mGy)			
0	81 (18.4)	11 (16.2)	Not applicable
1-99	193 (43.9)	14 (20.6)	
100-199	90 (20.5)	1 (1.5)	
200-499	43 (9.8)	11 (16.2)	
≥500	33 (7.5)	31 (45.6)	
Mean of exposed (± SD)	*247 (± 665)*	*1119 (± 1421)*	
Age at exposure (years)			
Unexposed	81 (18.4)	11 (16.2)	Not applicable
<5	139 (31.6)	27 (39.7)	
5-9	102 (23.2)	15 (22.1)	
≥10	118 (26.8)	15 (22.1)	
Mean of exposed (± SD)	*7.3 (± 5.1)*	*6.2 (± 4.6)*	

Thyroid surgery volume			
Hemithyroidectomy	58 (13.2)	0 (0.0)	Not available
Total thyroidectomy	365 (83.0)	0 (0.0)	
Unknown	17 (3.9)	68 (100.0)	
Pathologic T			
T1a	70 (15.9)	7 (10.3)	97 (29.8)
T1b	136 (30.9)	22 (32.4)	
T2	71 (16.1)	9 (13.2)	112 (34.4)
T3	163 (37.0)	30 (44.1)	106 (32.5)
T4	0 (0.0)	0 (0.0)	11 (3.4)
Pathologic N			
N0	262 (59.5)	39 (57.4)	178 (54.6)
N1	178 (40.5)	29 (42.6)	148 (45.4)
N1a	87 (19.8)	14 (20.6)	Not available
N1b	91 (20.7)	15 (22.1)	
Pathologic M			
M0	425 (96.6)	64 (94.1)	320 (98.2)
M1	15 (3.4)	4 (5.9)	6 (1.8)
Multifocal lesion			
No	362 (82.3)	61 (89.7)	Not available
Yes	78 (17.7)	7 (10.3)	
Primary lesion size (cm)			
≤1.0	93 (21.1)	10 (14.7)	Not available
>1.0-2.0	189 (43.0)	39 (57.4)	
>2.0-4.0	122 (27.7)	14 (20.6)	
>4.0	36 (8.2)	5 (7.4)	
Mean (± SD)	*2.0 (± 1.3)*	*1.9 (± 1.3)*	

Abbreviations: milligray (mGy), papillary thyroid carcinoma (PTC), standard deviation (SD), The Cancer Genome Atlas (TCGA).

* Restricted to individuals with known drivers who did not overlap with our primary study population from two previously published studies (see Supplementary Methods). Ricarte-Filho JC, et al. *J Clin Invest* 2013. Efanov AA, et al. *J Natl Cancer Inst* 2018.

† Restricted to individuals with known drivers and available pathology data; excluding individuals with known radiation exposure (see Supplementary Methods). The Cancer Genome Atlas Research Network. *Cell* 2014.

Supplementary Table S2. Distribution of PTC tumors by study population, pathologic TNM classification, and primary lesion size (See also Figure S1).

**Tumor characteristic**	**N0M0**	**N1M0**	**N0M1**	**N1M1**
	**N (%)***	**N (%)***	**N (%)***	**N (%)***
**Primary study population**				
Pathologic T				
T1a	51 (72.9)	18 (25.7)	0 (0.0)	1 (1.4)
T1b	99 (72.8)	35 (25.7)	0 (0.0)	2 (1.5)
T2	54 (76.1)	16 (22.5)	1 (1.4)	0 (0.0)
T3	57 (35.0)	95 (58.3)	0 (0.0)	11 (6.7)
Multifocal lesion				
No	220 (60.8)	131 (36.2)	0 (0.0)	11 (3.0)
Yes	41 (52.6)	33 (42.3)	1 (1.3)	3 (3.8)
Primary lesion size (cm)				
≤1.0	64 (68.8)	28 (30.1)	0 (0.0)	1 (1.1)
>1.0-2.0	118 (62.4)	69 (36.5)	0 (0.0)	2 (1.1)
>2.0-4.0	62 (50.8)	50 (41.0)	1 (0.8)	9 (7.4)
>4.0	17 (47.2)	17 (47.2)	0 (0.0)	2 (5.6)
**Other Chornobyl samples†**				
Pathologic T				
T1a	5 (71.4)	2 (28.6)	0 (0.0)	0 (0.0)
T1b	17 (77.3)	5 (22.7)	0 (0.0)	0 (0.0)
T2	4 (44.4)	5 (55.6)	0 (0.0)	0 (0.0)
T3	13 (43.3)	13 (43.3)	0 (0.0)	4 (13.3)
Multifocal lesion				
No	37 (60.7)	23 (37.7)	0 (0.0)	1 (1.6)
Yes	2 (28.6)	2 (28.6)	0 (0.0)	3 (42.9)
Primary lesion size (cm)				
≤1.0	6 (60.0)	4 (40.0)	0 (0.0)	0 (0.0)
>1.0-2.0	28 (71.8)	9 (23.1)	0 (0.0)	2 (5.1)
>2.0-4.0	4 (28.6)	9 (64.3)	0 (0.0)	1 (7.1)
>4.0	1 (20.0)	3 (60.0)	0 (0.0)	1 (20.0)
**TCGA‡**				
Pathologic T				
T1	65 (67.0)	31 (32.0)	0 (0.0)	1 (1.0)
T2	62 (55.4)	49 (43.8)	1 (0.9)	0 (0.0)
T3	46 (43.4)	58 (54.7)	2 (1.9)	0 (0.0)
T4	1 (9.1)	8 (72.7)	1 (9.1)	1 (9.1)

Abbreviations: papillary thyroid carcinoma (PTC), The Cancer Genome Atlas (TCGA).

* Row percentages.

† Restricted to non-overlapping individuals with our primary study population from two previously published studies: Ricarte-Filho JC, et al. *J Clin Invest* 2013. Efanov AA, et al. *J Natl Cancer Inst* 2018.

‡ The Cancer Genome Atlas Research Network. *Cell* 2014.

Supplementary Table S3. Results from logistic regression models predicting the occurrence of cLNM associated with patient, clinical, and molecular characteristics.

	All samples		*BRAF* mutation driver only	*RET* fusion driver only
**Characteristic**	**Univariate P***	**Adjusted for sex and age at PTC P***	**Adjusted for sex and age at PTC P***	**Adjusted for sex and age at PTC P***
Sex	9.2E-02	0.11	0.20	0.61
Age at PTC	4.7E-03	5.6E-03	0.28	0.44
Radiation dose	0.21	0.32	0.92	0.13
Tumor size	2.3E-03	9.1E-03	3.2E-02	1.2E-02
Multifocality	0.22	0.10	0.11	0.39
Driver type	2.2E-07	5.8E-06	NA	NA
Driver gene	9.9E-21	1.6E-19	NA	NA
Total SSVs	0.63	0.72	0.14	0.69
SNV	0.70	0.67	0.15	0.72
Doublet/triplet	0.10	0.31	0.96	0.89
Small indels	5.3E-02	0.35	0.26	0.18
Small insertions	1.0E-03	7.5E-03	0.87	0.19
Small deletions	0.28	1.0E+00	0.14	0.24
Indel:SNV ratio	0.54	0.55	0.68	8.9E-02
Insertion:SNV ratio	1.7E-02	7.6E-03	0.38	0.12
Deletion:SNV ratio	0.76	0.66	0.97	0.17
Clock (SBS1,SBS5)	0.21	0.97	1.7E-02	0.93
SBS1	5.4E-04	6.2E-03	0.21	0.47
SBS5	0.40	0.67	1.5E-02	1.00
APOBEC (SBS2,SBS13)	0.36	0.39	0.86	4.5E-01
SBS2	0.29	0.29	0.81	0.49
SBS13	0.26	0.31	0.97	0.96
SBS8	8.9E-02	0.53	0.34	4.9E-02
SBS18	0.73	0.27	0.65	0.39
SBS23	2.9E-04	4.0E-04	1.3E-02	0.85
Clock (ID1,ID5)	1.7E-02	9.7E-02	0.73	0.13
ID1	1.3E-04	2.3E-04	0.46	1.00
ID5	0.32	0.88	0.96	9.8E-02
End-joining DNA repair (ID6,ID8)	0.69	0.36	0.47	0.73
ID6	0.92	0.95	0.96	0.76
ID8	0.70	0.41	0.57	0.80
ID3	0.29	0.78	2.8E-02	0.64
ID4	0.53	0.68	0.60	0.38
Total confirmed SV calls	1.3E-03	3.4E-03	0.82	0.31
Complex	4.4E-02	3.2E-02	0.67	0.27
Translocations	0.10	0.28	0.46	0.47
Inversions	3.6E-02	0.18	0.39	0.66
Deletions	0.29	0.31	0.20	0.28
Tandem duplications	0.77	0.82	0.19	0.68
Total confirmed SV events	2.1E-03	9.7E-03	0.88	0.65
Simple/balanced	5.8E-04	7.5E-03	0.78	0.26
Complex	0.10	7.5E-02	0.70	0.65

Simple/unbalanced	0.89	0.75	0.41	0.90
Simple/balanced EJ	1.2E-03	2.2E-02	0.69	0.20
Non-simple/balanced EJ	0.15	0.16	0.87	0.95
Fraction of genome altered	1.1E-03	4.3E-04	0.39	0.37
Total SCNAs	0.13	0.11	0.44	0.51
Deletions	0.86	0.79	7.0E-02	0.90
Gains	1.6E-02	1.0E-02	0.83	0.30
CNLOH	0.16	0.14	5.1E-02	0.27
22q deletion	5.8E-02	5.1E-02	4.2E-02	0.42

Abbreviations: apolipoprotein B mRNA editing enzyme, catalytic polypeptide-like (APOBEC), cervical lymph node metastasis (cLNM), copy-neutral loss of heterozygosity (CNLOH), end-joining (EJ), insertion-deletion (ID), not applicable (NA), simple somatic variant (SSV), single base substitution (SBS), single nucleotide variant (SNV), somatic copy number alteration (SCNA), structural variant (SV).

* P-values are two-sided P-value calculated using likelihood ratio tests, comparing model fit with and without the variable of interest. Regression models are described in the Methods.

Supplementary Table S4. Distribution of PTC tumors by study population, occurrence of cLNM, and PTC driver (See also Figure 1 and Figure S3).

	Primary study population (N=428)*			Other Chornobyl samples (N=68)†			TCGA (N=322)‡		
	No cLNM	cLNM		No cLNM	cLNM		No cLNM	cLNM	
**Driver§**	**N (%)‖**	**N (%)‖**	**$P_{heterogeneity}$**	**N (%)‖**	**N (%)‖**	**$P_{heterogeneity}$**	**N (%)‖**	**N (%)‖**	**$P_{heterogeneity}$**
Driver type			5.8E-06			0.81			0.44
Fusion	79 (44.9%)	97 (55.1%)		25 (52.1%)	23 (47.9%)		28 (49.1%)	29 (50.9%)	
Mutation	176 (69.8%)	76 (30.2%)		14 (70.0%)	6 (30.0%)		146 (55.1%)	119 (44.9%)	
Driver gene			1.6E-19			0.39			1.4E-05
BRAF mutation	121 (62.4%)	73 (37.6%)		10 (62.5%)	6 (37.5%)		106 (49.8%)	107 (50.2%)	
Other mutation	55 (94.8%)	3 (5.2%)		4 (100.0%)	0 (0.0%)		40 (76.9%)	12 (23.1%)	
RAS	41 (95.3%)	2 (4.7%)		3 (100.0%)	0 (0.0%)		35 (77.8%)	10 (22.2%)	
APC	2 (100.0%)	0 (0.0%)		Not observed			Not observed		
DICER1	3 (100.0%)	0 (0.0%)		Not observed			Not observed		
NFE2L2	1 (50.0%)	1 (50.0%)		Not observed			Not observed		
TSC	2 (100.0%)	0 (0.0%)		Not observed			Not observed		
TSHR	6 (100.0%)	0 (0.0%)		1 (100.0%)	0 (0.0%)		1 (100.0%)	0 (0.0%)	
CHEK2	Not observed			Not observed			0 (0.0%)	1 (100.0%)	
EIF1AX	Not observed			Not observed			4 (80.0%)	1 (20.0%)	
RET fusion	21 (28.8%)	52 (71.2%)		13 (44.8%)	16 (55.2%)		5 (21.7%)	18 (78.3%)	
Other *RTK* fusion	23 (35.9%)	41 (64.1%)		8 (66.7%)	4 (33.3%)		5 (50.0%)	5 (50.0%)	
ALK	4 (33.3%)	8 (66.7%)		3 (75.0%)	1 (25.0%)		2 (66.7%)	1 (33.3%)	
LTK	1 (33.3%)	2 (66.7%)		Not observed			0 (0.0%)	1 (100.0%)	
NTRK1	2 (15.4%)	11 (84.6%)		1 (100.0%)	0 (0.0%)		0 (0.0%)	2 (100.0%)	
NTRK3	16 (44.4%)	20 (55.6%)		4 (57.1%)	3 (42.9%)		3 (75.0%)	1 (25.0%)	
Other fusion	35 (89.7%)	4 (10.3%)		4 (57.1%)	3 (42.9%)		18 (75.0%)	6 (25.0%)	
BRAF	16 (80.0%)	4 (20.0%)		3 (50.0%)	3 (50.0%)		6 (60.0%)	4 (40.0%)	
IGF2/IGF2BP3	6 (100.0%)	0 (0.0%)		Not observed			6 (100.0%)	0 (0.0%)	
PPARG	13 (100.0%)	0 (0.0%)		1 (100.0%)	0 (0.0%)		3 (75.0%)	1 (25.0%)	
FGFR2	Not observed			Not observed			1 (100.0%)	0 (0.0%)	
MET	Not observed			Not observed			0 (0.0%)	1 (100.0%)	
PTEN	Not observed			Not observed			2 (100.0%)	0 (0.0%)	

Abbreviations: cervical lymph node metastasis (cLNM), papillary thyroid carcinoma (PTC), The Cancer Genome Atlas (TCGA).

* Analyses exclude N=1 individual with distant metastases only and N=11 individuals without a final designated driver, of which 5 (45.4%) had cLNM. $P_{heterogeneity}$ calculated using a two-sided P-value generated using likelihood ratio tests, comparing model fit with and without the variable of interest.

† Restricted to non-overlapping individuals with our primary study population from two previously published studies: Ricarte-Filho JC, et al. *J Clin Invest* 2013. Efanov AA, et al. *J Natl Cancer Inst* 2018.

‡ The Cancer Genome Atlas Research Network. *Cell* 2014.

§ Tumors were characterized by their final designated driver in our primary study population, which required observed alterations to be recurrent in our dataset (See Methods).

‖ Row percentages.

Supplementary Table S5. Distribution of cLNM among PTC tumors with fusion drivers.

Driver	No cLNM	cLNM Total	cLNM N1a	cLNM N1b
	N (%)*	N (%)*	N (%)*	N (%)*
RET fusion	21 (28.8%)	52 (71.2%)	21 (28.8%)	31 (42.5%)
NCOA4-RET	3 (20.0%)	12 (80.0%)	3 (20.0%)	9 (60.0%)
CCDC6-RET	14 (35.0%)	26 (65.0%)	10 (25.0%)	16 (40.0%)
RET -Other	4 (22.2%)	14 (77.8%)	8 (44.4%)	6 (33.3%)
ALK fusion	4 (33.3%)	8 (66.7%)	3 (25.0%)	5 (41.7%)
ALK-STRN	2 (25.0%)	6 (75.0%)	2 (25.0%)	4 (50.0%)
ALK -Other	2 (50.0%)	2 (50.0%)	1 (25.0%)	1 (25.0%)
LTK fusion	1 (33.3%)	2 (66.7%)	2 (66.7%)	0 (0.0%)
NTRK1 fusion	2 (15.4%)	11 (84.6%)	3 (23.1%)	8 (61.5%)
NTRK1-TPM3	0 (0.0%)	4 (100.0%)	0 (0.0%)	4 (100.0%)
NTRK1-TPR	1 (20.0%)	4 (80.0%)	1 (20.0%)	3 (60.0%)
NTRK1 -Other	1 (25.0%)	3 (75.0%)	1 (25.0%)	2 (50.0%)
NTRK3 fusion	16 (44.4%)	20 (55.6%)	7 (19.4%)	13 (36.1%)
ETV6-NTRK3	15 (46.9%)	17 (53.1%)	6 (18.8%)	11 (34.4%)
NTRK3 -Other	1 (25.0%)	3 (75.0%)	1 (25.0%)	2 (50.0%)
BRAF fusion	16 (80.0%)	4 (20.0%)	4 (20.0%)	0 (0.0%)
AGK-BRAF	5 (83.3%)	1 (16.7%)	1 (16.7%)	0 (0.0%)
BRAF-SND1	3 (75.0%)	1 (25.0%)	1 (25.0%)	0 (0.0%)
BRAF -Other	7 (77.8%)	2 (22.2%)	2 (22.2%)	0 (0.0%)
BRAF large deletion	1 (100.0%)	0 (0.0%)	0 (0.0%)	0 (0.0%)
IGF2/IGF2BP3 structural variant	6 (100.0%)	0 (0.0%)	0 (0.0%)	0 (0.0%)
PPARG fusion	13 (100.0%)	0 (0.0%)	0 (0.0%)	0 (0.0%)

Abbreviations: cervical lymph node metastasis (cLNM), papillary thyroid carcinoma (PTC).

* Row percentages.

Supplementary Table S6. Distribution of driver, age at PTC, and presence of cervical lymph node metastases (cLNM) at diagnosis among the N=428 PTC tumors in our primary study population with a final designated driver.

**Driver**<br>**Age at PTC**	**No cLNM**<br>**N (%)***	**cLNM**<br>**N (%)***
BRAF mutation		
<20	13 (76.5%)	4 (23.5%)
20-24	12 (42.9%)	16 (57.1%)
25-29	28 (60.9%)	18 (39.1%)
30-34	44 (64.7%)	24 (35.3%)
≥35	24 (68.6%)	11 (31.4%)
Other mutation		
<20	6 (100.0%)	0 (0.0%)
20-24	13 (100.0%)	0 (0.0%)
25-29	15 (88.2%)	2 (11.8%)
30-34	12 (100.0%)	0 (0.0%)
≥35	9 (90.0%)	1 (10.0%)
RET fusion		
<20	5 (23.8%)	16 (76.2%)
20-24	5 (27.8%)	13 (72.2%)
25-29	7 (35.0%)	13 (65.0%)
30-34	3 (33.3%)	6 (66.7%)
≥35	1 (20.0%)	4 (80.0%)
Other *RTK* fusion		
<20	5 (35.7%)	9 (64.3%)
20-24	2 (22.2%)	7 (77.8%)
25-29	6 (31.6%)	13 (68.4%)
30-34	9 (50.0%)	9 (50.0%)
≥35	1 (25.0%)	3 (75.0%)
Other fusion		
<20	6 (100.0%)	0 (0.0%)
20-24	6 (100.0%)	0 (0.0%)
25-29	7 (77.8%)	2 (22.2%)
30-34	13 (86.7%)	2 (13.3%)
≥35	3 (100.0%)	0 (0.0%)

Abbreviations: cervical lymph node metastasis (cLNM), papillary thyroid carcinoma (PTC), standard deviation (SD).
* Indicates row percentages.

Supplementary Table S7. Relationship of patient and pathologic characteristics to occurrence of cLNM in a pooled analysis with previous Chornobyl studies (See Tables S1-S2 and S4).

	***BRAF* mutation**			***RET* fusion**		
	**cLNM**			**cLNM**		
**Characteristic**	**No**	**Yes**	**OR (95%CI)***	**No**	**Yes**	**OR (95%CI)***
Sex						
Female	109	57	1.0 (referent)	22	50	1.0 (referent)
Male	22	22	1.6 (0.8 , 3.4)	12	18	1.2 (0.4 , 3.7)
Age at PTC (years)						
<25	30	24	1.0 (referent)	18	43	1.0 (referent)
25-29	29	20	0.7 (0.3 , 1.6)	12	14	0.5 (0.1 , 1.5)
≥30	72	35	**0.4 (0.2 , 0.9)**	4	11	1.9 (0.4 , 9.3)
P_{trend}			0.053			0.41
Radiation dose (mGy)						
0	26	13	1.0 (referent)	5	15	1.0 (referent)
1-99	68	44	2.2 (0.8 , 5.9)	10	19	0.6 (0.1 , 3.6)
100-199	19	17	**3.2 (1.1 , 9.8)**	4	10	1.7 (0.3 , 11.1)
≥200	18	5	0.9 (0.2 , 3.1)	15	24	0.8 (0.2 , 3.4)
P_{trend}			0.28			0.64
Multifocal lesion						
No	109	60	1.0 (referent)	30	59	1.0 (referent)
Yes	22	19	1.8 (0.9 , 3.9)	4	9	1.0 (0.2 , 4.7)
Primary lesion size (cm)						
≤1.0	38	17	1.0 (referent)	6	6	1.0 (referent)
>1.0-2.0	69	34	1.1 (0.5 , 2.2)	25	25	1.2 (0.3 , 4.8)
>2.0	24	28	**2.6 (1.1 , 6.2)**	3	37	**17.3 (2.8 , 106)**
P_{trend}			**1.9E-03**			**8.7E-06**

Abbreviations: cervical lymph node metastasis (cLNM), confidence interval (CI), odds ratio (OR), papillary thyroid carcinoma (PTC).

Bolded font represents P<0.05. P_{trend} represents a two-sided P-value calculated using likelihood ratio tests, comparing model fit with and without the variable of interest.

* Multivariable logistic regression model includes all patient and pathologic characteristics in the table.

Supplementary Table S8. Relationship of patient and pathologic characteristics to occurrence of cLNM in a pooled analysis with previous Chornobyl studies and TCGA (See Tables S1-S2 and S4).

	***BRAF* mutation**			***RET* fusion**		
	**cLNM**			**cLNM**		
**Characteristic**	**No**	**Yes**	**OR (95%CI)***	**No**	**Yes**	**OR (95%CI)***
Sex						
Female	193	130	1.0 (referent)	27	65	1.0 (referent)
Male	44	56	**1.7 (1.1 , 2.8)**	12	21	0.8 (0.3 , 2.1)
Age at PTC (years)						
<25	34	34	1.0 (referent)	21	45	1.0 (referent)
25-34	93	68	0.8 (0.4 , 1.4)	16	25	0.8 (0.3 , 2.2)
35-44	47	33	0.7 (0.3 , 1.4)			
45-54	32	23	0.6 (0.3 , 1.3)	2	16	2.5 (0.8 , 7.7)
≥55	31	28	0.6 (0.3 , 1.3)			
P_{trend}			0.27			0.38
Pathologic T						
T1	131	54	1.0 (referent)	24	19	1.0 (referent)
T2	48	39	**1.9 (1.1 , 3.3)**	4	16	**4.5 (1.2 , 16.3)**
T3/4	58	93	**3.9 (2.4 , 6.2)**	11	51	**6.4 (2.5 , 16.1)**

Abbreviations: cervical lymph node metastasis (cLNM), confidence interval (CI), odds ratio (OR), papillary thyroid carcinoma (PTC), The Cancer Genome Atlas (TCGA).
Bolded font represents P<0.05. P_{trend} represents a two-sided P-value calculated using likelihood ratio tests, comparing model fit with and without the variable of interest.
* Multivariable logistic regression model includes all patient and pathologic characteristics in the table.

Supplementary Table S9. Distribution of PTC driver among cLNM and PT samples by platform.

	Total cLNM samples		WGS			mRNA-seq			miRNA-seq			DNA methylation			Relative telomere length		
	**Unavailable**	**Included**	**cLNM**	**Paired PT**	**Matched**	**cLNM**	**Paired PT**	**Matched**	**cLNM**	**Paired PT**	**Matched**	**cLNM**	**Paired PT**	**Matched**	**cLNM**	**Paired PT**	**Matched**
**PTC driver**	**N (%)***	**N (%)***	**N (%)**	**N (%)**	**N (%)**	**N (%)**	**N (%)**	**N (%)**	**N (%)**	**N (%)**	**N (%)**	**N (%)**	**N (%)**	**N (%)**	**N (%)**	**N (%)**	**N (%)**
BRAF mutation	63 (86.3%)	10 (13.7%)	9 (20.0%)	8 (19.5%)	18 (21.2%)	9 (19.6%)	9 (20.5%)	18 (20.2%)	10 (23.3%)	10 (23.8%)	18 (22.2%)	8 (18.6%)	5 (13.9%)	16 (18.6%)	9 (20.9%)	8 (20.5%)	18 (22.2%)
Other mutation	3 (100.0%)	0 (0.0%)	0 (0.0%)	0 (0.0%)	0 (0.0%)	0 (0.0%)	0 (0.0%)	0 (0.0%)	0 (0.0%)	0 (0.0%)	0 (0.0%)	0 (0.0%)	0 (0.0%)	0 (0.0%)	0 (0.0%)	0 (0.0%)	0 (0.0%)
RAS	2 (100.0%)	0 (0.0%)	0 (0.0%)	0 (0.0%)	0 (0.0%)	0 (0.0%)	0 (0.0%)	0 (0.0%)	0 (0.0%)	0 (0.0%)	0 (0.0%)	0 (0.0%)	0 (0.0%)	0 (0.0%)	0 (0.0%)	0 (0.0%)	0 (0.0%)
NFE2L2	1 (100.0%)	0 (0.0%)	0 (0.0%)	0 (0.0%)	0 (0.0%)	0 (0.0%)	0 (0.0%)	0 (0.0%)	0 (0.0%)	0 (0.0%)	0 (0.0%)	0 (0.0%)	0 (0.0%)	0 (0.0%)	0 (0.0%)	0 (0.0%)	0 (0.0%)
RET fusion	34 (65.4%)	18 (34.6%)	17 (37.8%)	15 (36.6%)	34 (40.0%)	18 (39.1%)	16 (36.4%)	36 (40.4%)	16 (37.2%)	15 (35.7%)	32 (39.5%)	17 (39.5%)	15 (41.7%)	34 (39.5%)	16 (37.2%)	14 (35.9%)	32 (39.5%)
Other *RTK* fusion	23 (56.1%)	18 (43.9%)	18 (40.0%)	18 (43.9%)	31 (36.5%)	18 (39.1%)	18 (40.9%)	33 (37.1%)	16 (37.2%)	16 (38.1%)	29 (35.8%)	17 (39.5%)	16 (44.4%)	34 (39.5%)	17 (39.5%)	17 (43.6%)	29 (35.8%)
ALK	3 (37.5%)	5 (62.5%)	5 (11.1%)	5 (12.2%)	6 (7.1%)	5 (10.9%)	5 (11.4%)	7 (7.9%)	5 (11.6%)	5 (11.9%)	7 (8.6%)	4 (9.3%)	4 (11.1%)	8 (9.3%)	5 (11.6%)	5 (12.8%)	6 (7.4%)
LTK	1 (50.0%)	1 (50.0%)	1 (2.2%)	1 (2.4%)	2 (2.4%)	1 (2.2%)	1 (2.3%)	2 (2.2%)	1 (2.3%)	1 (2.4%)	2 (2.5%)	1 (2.3%)	1 (2.8%)	2 (2.3%)	1 (2.3%)	1 (2.6%)	2 (2.5%)
NTRK1	7 (63.6%)	4 (36.4%)	4 (8.9%)	4 (9.8%)	7 (8.2%)	4 (8.7%)	4 (9.1%)	8 (9.0%)	3 (7.0%)	3 (7.1%)	6 (7.4%)	4 (9.3%)	4 (11.1%)	8 (9.3%)	4 (9.3%)	4 (10.3%)	7 (8.6%)
NTRK3	12 (60.0%)	8 (40.0%)	8 (17.8%)	8 (19.5%)	16 (18.8%)	8 (17.4%)	8 (18.2%)	16 (18.0%)	7 (16.3%)	7 (16.7%)	14 (17.3%)	8 (18.6%)	7 (19.4%)	16 (18.6%)	7 (16.3%)	7 (17.9%)	14 (17.3%)
BRAF fusion	3 (75.0%)	1 (25.0%)	1 (2.2%)	0 (0.0%)	2 (2.4%)	1 (2.2%)	1 (2.3%)	2 (2.2%)	1 (2.3%)	1 (2.4%)	2 (2.5%)	1 (2.3%)	0 (0.0%)	2 (2.3%)	1 (2.3%)	0 (0.0%)	2 (2.5%)
Unknown	5 (100.0%)	0 (0.0%)	0 (0.0%)	0 (0.0%)	0 (0.0%)	0 (0.0%)	0 (0.0%)	0 (0.0%)	0 (0.0%)	0 (0.0%)	0 (0.0%)	0 (0.0%)	0 (0.0%)	0 (0.0%)	0 (0.0%)	0 (0.0%)	0 (0.0%)

Abbreviations: cervical lymph node metastasis (cLNM), papillary thyroid carcinoma (PTC), primary tumor (PT), whole genome sequencing (WGS).

* Indicates row percentages. All other percentages are column percentages.

Supplementary Table S10. Distribution of available cLNM samples by sex, age at PTC, and radiation dose

Characteristic	Unavailable N (%)*	Included N (%)*
Sex		
Female	96 (75.0%)	32 (25.0%)
Male	35 (70.0%)	15 (30.0%)
Age at PTC (years)		
<15	6 (66.7%)	3 (33.3%)
15-19	13 (65.0%)	7 (35.0%)
20-24	25 (69.4%)	11 (30.6%)
25-29	40 (76.9%)	12 (23.1%)
30-34	31 (75.6%)	10 (24.4%)
35-39	16 (84.2%)	3 (15.8%)
≥40	0 (0.0%)	1 (100.0%)
Mean (± SD)	*27.3 (± 6.4)*	*25.6 (± 6.8)*
Radiation dose (mGy)		
0	20 (69.0%)	9 (31.0%)
1-99	52 (71.2%)	21 (28.8%)
100-199	32 (76.2%)	10 (23.8%)
200-499	16 (88.9%)	2 (11.1%)
≥500	11 (68.8%)	5 (31.3%)
Mean of exposed (± SD)	*190 (± 231)*	*188 (± 266)*

Abbreviations: cervical lymph node metastasis (cLNM), papillary thyroid carcinoma (PTC), standard deviation (SD).
* Indicates row percentages.

Supplementary Table S11. Distribution of genomic characteristics by type among cLNM and PT samples.

	All samples		Paired cLNM-PT samples	
	PT (N=126)	cLNM (N=45)	PT (N=41)	cLNM (N=41)
**Mutation**	**N (%)**	**N (%)**	**N (%)**	**N (%)**
Total SSVs	98655 (100.0%)	34031 (100.0%)	32801 (100.0%)	30315 (100.0%)
By type				
SNV	91927 (93.2%)	31508 (92.6%)	30544 (93.1%)	28016 (92.4%)
Doublet/triplet	412 (0.4%)	135 (0.4%)	132 (0.4%)	121 (0.4%)
Small insertion/deletion	6316 (6.4%)	2388 (7.0%)	2125 (6.5%)	2178 (7.2%)
Deletion	4604 (4.7%)	1722 (5.1%)	1549 (4.7%)	1559 (5.1%)
Insertion	1712 (1.7%)	666 (2.0%)	576 (1.8%)	619 (2.0%)
SBS signatures				
Clock	62885 (68.4%)	21327 (67.7%)	20388 (66.7%)	18873 (67.4%)
SBS1	8705 (9.5%)	3091 (9.8%)	2819 (9.2%)	2803 (10.0%)
SBS5	54180 (58.9%)	18236 (57.9%)	17569 (57.5%)	16070 (57.4%)
APOBEC	14349 (15.6%)	5463 (17.3%)	6026 (19.7%)	5038 (18.0%)
SBS2	6980 (7.6%)	2542 (8.1%)	2595 (8.5%)	2351 (8.4%)
SBS13	7369 (8.0%)	2921 (9.3%)	3431 (11.2%)	2687 (9.6%)
SBS8	12474 (13.6%)	4076 (12.9%)	3546 (11.6%)	3463 (12.4%)
SBS18	1006 (1.1%)	327 (1.0%)	304 (1.0%)	327 (1.2%)
SBS23	1213 (1.3%)	315 (1.0%)	280 (0.9%)	315 (1.1%)
ID signatures				
Clock	3267 (51.7%)	1302 (54.5%)	1179 (55.5%)	1183 (54.3%)
ID1	758 (12.0%)	298 (12.5%)	283 (13.3%)	281 (12.9%)
ID5	2509 (39.7%)	1004 (42.0%)	896 (42.2%)	902 (41.4%)
End-joining	1289 (20.4%)	530 (22.2%)	458 (21.6%)	500 (23.0%)
ID6	176 (2.8%)	46 (1.9%)	40 (1.9%)	35 (1.6%)
ID8	1113 (17.6%)	484 (20.3%)	418 (19.7%)	465 (21.3%)
ID3	1316 (20.8%)	403 (16.9%)	361 (17.0%)	372 (17.1%)
ID4	444 (7.0%)	153 (6.4%)	127 (6.0%)	123 (5.6%)
Confirmed structural variants				
0	22 (17.5%)	10 (22.2%)	8 (19.5%)	9 (22.0%)
1	62 (49.2%)	19 (42.2%)	17 (41.5%)	16 (39.0%)
2	20 (15.9%)	11 (24.4%)	6 (14.6%)	11 (26.8%)
≥3	22 (17.5%)	5 (11.1%)	10 (24.4%)	5 (12.2%)
Total SCNAs				
0	75 (59.5%)	28 (62.2%)	25 (61.0%)	24 (58.5%)
1	35 (27.8%)	10 (22.2%)	11 (26.8%)	10 (24.4%)
2	8 (6.3%)	3 (6.7%)	3 (7.3%)	3 (7.3%)
≥3	8 (6.3%)	4 (8.9%)	2 (4.9%)	4 (9.8%)
Total SCNA deletions				
0	83 (65.9%)	30 (66.7%)	27 (65.9%)	26 (63.4%)
1	31 (24.6%)	12 (26.7%)	11 (26.8%)	12 (29.3%)
≥2	12 (9.5%)	3 (6.7%)	3 (7.3%)	3 (7.3%)
Total SCNA gains				
0	113 (89.7%)	39 (86.7%)	39 (95.1%)	35 (85.4%)
1	11 (8.7%)	4 (8.9%)	1 (2.4%)	4 (9.8%)
≥2	2 (1.6%)	2 (4.4%)	1 (2.4%)	2 (4.9%)
22q deletion				
No	115 (91.3%)	43 (95.6%)	40 (97.6%)	39 (95.1%)
Yes	11 (8.7%)	2 (4.4%)	1 (2.4%)	2 (4.9%)
	Mean (± SD)	Mean (± SD)	Mean (± SD)	Mean (± SD)
Mutation density per Mb	0.27 (± 0.13)	0.27 (± 0.11)	0.28 (± 0.12)	0.26 (± 0.10)
Fraction of genome altered	0.30% (± 0.80%)	0.44% (± 1.35%)	0.17% (± 0.35%)	0.48% (± 1.41%)

Abbreviations: apolipoprotein B mRNA editing enzyme, catalytic polypeptide-like (APOBEC), cervical lymph node metastasis (cLNM), insertion-deletion (ID), megabase (Mb), primary tumor (PT), single base substitution (SBS), single nucleotide variant (SNV), simple somatic variant (SSV), somatic copy number alteration (SCNA), standard deviation (SD).

Supplementary Table S12. Results from regression models comparing genomic characteristics of cLNM and PT.

		Primary analysis	Sensitivity analyses		
**Characteristic**	**Regression model**	**Adjusted for sex and age at PTC P***	**Adjusted for sex, age at PTC, and driver P***	**Adjusted for sex, age at PTC, and radiation dose P***	**Restricted to paired cLNM-PT samples P***
Total SSVs	Linear	0.64	0.42	0.61	0.40
SNV	Linear	0.60	0.43	0.56	0.36
Doublet/triplet	Proportional odds	0.39	0.45	0.41	0.64
Small indels	Linear	0.48	0.49	0.32	0.84
Small insertions	Linear	0.24	0.63	0.20	0.47
Small deletions	Linear	0.64	0.42	0.45	1.00
Indel:SNV ratio	Linear	0.21	0.65	0.081	0.22
Insertion:SNV ratio	Linear	0.044	0.84	0.029	0.064
Deletion:SNV ratio	Linear	0.44	0.50	0.22	0.43
Clock (SBS1,SBS5)	Linear	0.35	0.55	0.34	0.35
SBS1	Linear	0.92	0.34	0.92	0.92
SBS5	Linear	0.31	0.57	0.30	0.30
APOBEC (SBS2,SBS13)	Proportional odds	0.081	0.062	0.094	0.47
SBS2	Proportional odds	0.12	0.091	0.13	0.70
SBS13	Proportional odds	0.093	0.055	0.12	0.68
SBS8	Linear	0.44	0.51	0.48	0.89
SBS18	Proportional odds	0.60	0.61	0.60	0.77
SBS23	Proportional odds	0.46	0.45	0.51	0.76
Clock (ID1,ID5)	Linear	0.24	0.62	0.15	1.00
ID1	Linear	0.44	0.51	0.45	1.00
ID5	Linear	0.32	0.56	0.21	1.00
End-joining DNA repair (ID6,ID8)	Linear	0.40	0.53	0.25	0.71
ID6	Proportional odds	0.67	0.69	0.57	0.88
ID8	Linear	0.32	0.58	0.17	0.68
ID3	Linear	0.30	0.59	0.30	0.89
ID4	Proportional odds	0.84	0.94	0.86	0.93
Total confirmed SV calls	Proportional odds	0.19	0.046	0.20	0.69
Complex	Proportional odds	0.18	0.10	0.15	0.71
Translocations	Logistic	0.15	0.12	0.13	1.00
Inversions	Logistic	0.12	0.08	0.14	0.77
Deletions	Logistic	0.87	0.91	0.87	0.80
Tandem duplications	Logistic	0.68	0.70	0.66	1.00
Total confirmed SV events	Proportional odds	0.73	0.54	0.77	0.61
Simple/balanced	Proportional odds	0.88	1.00	0.72	0.68
Complex	Proportional odds	0.28	0.22	0.26	0.73
Simple/unbalanced	Proportional odds	0.72	0.90	0.73	0.93
Simple/balanced EJ	Logistic	0.93	0.88	0.74	0.82
Non-simple/balanced EJ	Proportional odds	0.38	0.20	0.36	0.75
Fraction of genome altered	Linear	0.40	0.53	0.42	0.17
Total SCNAs	Proportional odds	0.97	1.00	0.94	0.69
Deletions	Proportional odds	0.86	0.84	0.80	0.83
Gains	Proportional odds	0.53	0.52	0.53	0.13
CNLOH	Logistic	0.12	0.12	0.11	0.093
22q deletion	Logistic	0.33	0.33	0.31	0.55
Relative telomere length	Linear	0.014	0.015	0.014	0.24

Abbreviations: apolipoprotein B mRNA editing enzyme, catalytic polypeptide-like (APOBEC), cervical lymph node metastasis (cLNM), copy-neutral loss of heterozygosity (CNLOH), end-joining (EJ), insertion-deletion (ID), megabase (Mb), papillary thyroid carcinoma (PTC), primary tumor (PT), simple somatic variant (SSV), single base substitution (SBS), single nucleotide variant (SNV), somatic copy number alteration (SCNA), structural variant (SV).

* P-values are two-sided P-values calculated using likelihood ratio tests, comparing model fit with and without the variable of interest.

Supplementary Table S13. Distribution of simple somatic variants by type and cancer cell fraction among paired cLNM-PT samples.

	Count		% of mutations	
	**Mean (± SD)**	**Range**	**Mean (± SD)**	**Range**
**SNVs**				
Total				
PT private	416.8 (± 344.4)	42 - 1387	35.0% (± 16.2%)	8.3% - 63.0%
cLNM private	355.3 (± 274.0)	39 - 1120	30.7% (± 15.7%)	5.0% - 65.5%
shared	323.5 (± 161.7)	72 - 645	34.3% (± 19.5%)	7.8% - 75.9%
By clonality				
clonal PT private	78.7 (± 181.0)	0 - 800	5.6% (± 9.7%)	0.0% - 41.1%
subclonal PT private	338.1 (± 266.8)	42 - 1308	30.4% (± 14.3%)	5.7% - 58.5%
clonal cLNM private	60.0 (± 78.8)	0 - 325	6.0% (± 7.4%)	0.0% - 29.8%
subclonal cLNM private	295.4 (± 271.0)	8 - 1089	25.4% (± 17.2%)	1.0% - 64.4%
clonal shared	270.4 (± 147.8)	41 - 615	29.4% (± 18.0%)	5.6% - 72.7%
subclonal shared	28.2 (± 37.3)	0 - 170	3.2% (± 4.2%)	0.0% - 15.4%
**Small insertions**				
Total				
PT private	6.4 (± 6.0)	1 - 37	28.0% (± 16.8%)	3.8% - 68.5%
cLNM private	7.4 (± 4.0)	1 - 15	34.6% (± 15.3%)	7.1% - 63.6%
shared	7.7 (± 4.4)	1 - 20	37.4% (± 17.4%)	5.9% - 76.9%
By clonality				
clonal PT private	2.5 (± 4.2)	0 - 25	10.3% (± 12.7%)	0.0% - 55.6%
subclonal PT private	3.9 (± 3.2)	0 - 12	18.1% (± 12.6%)	0.0% - 45.8%
clonal cLNM private	2.9 (± 2.7)	0 - 10	12.9% (± 11.0%)	0.0% - 41.2%
subclonal cLNM private	4.5 (± 3.7)	0 - 13	22.2% (± 18.4%)	0.0% - 65.0%
clonal shared	7.2 (± 4.2)	1 - 19	35.9% (± 16.9%)	5.9% - 73.1%
subclonal shared	0.1 (± 0.4)	0 - 2	0.6% (± 1.7%)	0.0% - 9.1%
**Small deletions**				
Total				
PT private	19.7 (± 14.1)	3 - 65	33.1% (± 13.0%)	7.3% - 65.7%
cLNM private	20.0 (± 12.3)	2 - 67	35.9% (± 14.8%)	11.8% - 85.7%
shared	17.7 (± 14.7)	0 - 84	31.0% (± 15.5%)	0.0% - 70.6%
By clonality				
clonal PT private	8.6 (± 7.8)	0 - 38	14.4% (± 8.7%)	0.0% - 38.4%
subclonal PT private	11.1 (± 9.1)	1 - 35	19.6% (± 12.2%)	3.3% - 46.7%
clonal cLNM private	8.8 (± 7.2)	0 - 32	15.4% (± 10.7%)	0.0% - 42.7%
subclonal cLNM private	11.2 (± 10.2)	0 - 38	21.4% (± 17.9%)	0.0% - 81.0%
clonal shared	15.7 (± 14.5)	0 - 82	27.4% (± 14.7%)	0.0% - 68.8%
subclonal shared	0.8 (± 1.2)	0 - 5	1.9% (± 3.6%)	0.0% - 20.0%

Abbreviations: cervical lymph node metastasis (cLNM), primary tumor (PT), single nucleotide variant (SNV), standard deviation (SD).
