# Supplementary material for: Genomic characterization of cervical lymph node metastases in papillary thyroid carcinoma following the Chornobyl accident
Source: Nat Commun. 2024 Jun 13;15:5053. doi: 10.1038/s41467-024-49292-z (PMC11176192; doi:10.1038/s41467-024-49292-z)
Supplement: Supplementary file 3 — Description of Additional Supplementary Files [file 41467_2024_49292_MOESM3_ESM.pdf]

Description of Additional Supplementary Files

Supplementary Data 1. Sample IDs included in each analysis.

Supplementary Data 2. Analytic data comparing tumors with and without cLNM

Supplementary Data 3. Analytic data comparing cLNM and PT samples.

Supplementary Data 4. Differential mRNA expression modeling results comparing cLNM to PT expression.

Supplementary Data 5. Differential mRNA expression modeling results comparing cLNM to PT expression.

Supplementary Data 6. Differential miRNA expression modeling results comparing cLNM to PT expression.

Supplementary Data 7. Differential methylation modeling results comparing cLNM to PT expression.

Supplementary Data 8. miRNA excluded from analysis due to lack of expression in our dataset
