# Supplementary material for: Genomic characterization of cervical lymph node metastases in papillary thyroid carcinoma following the Chornobyl accident
Source: Nat Commun. 2024 Jun 13;15:5053. doi: 10.1038/s41467-024-49292-z (PMC11176192; doi:10.1038/s41467-024-49292-z)
Supplement: Supplementary file 5 — Reporting Summary [file 41467_2024_49292_MOESM5_ESM.pdf]

nature portfolio

Corresponding author(s): Lindsay M. Morton

Last updated by author(s): Aug 23, 2023

Reporting Summary

Nature Portfolio wishes to improve the reproducibility of the work that we publish. This form provides structure for consistency and transparency in reporting. For further information on Nature Portfolio policies, see our Editorial Policies and the Editorial Policy Checklist.

Statistics

For all statistical analyses, confirm that the following items are present in the figure legend, table legend, main text, or Methods section.

n/a	Confirmed	
☐	☒	The exact sample size (*n*) for each experimental group/condition, given as a discrete number and unit of measurement
☐	☒	A statement on whether measurements were taken from distinct samples or whether the same sample was measured repeatedly
☐	☒	The statistical test(s) used AND whether they are one- or two-sided<br>*Only common tests should be described solely by name; describe more complex techniques in the Methods section.*
☐	☒	A description of all covariates tested
☐	☒	A description of any assumptions or corrections, such as tests of normality and adjustment for multiple comparisons
☐	☒	A full description of the statistical parameters including central tendency (e.g. means) or other basic estimates (e.g. regression coefficient) AND variation (e.g. standard deviation) or associated estimates of uncertainty (e.g. confidence intervals)
☐	☒	For null hypothesis testing, the test statistic (e.g. *F*, *t*, *r*) with confidence intervals, effect sizes, degrees of freedom and *P* value noted<br>*Give P values as exact values whenever suitable.*
☒	☐	For Bayesian analysis, information on the choice of priors and Markov chain Monte Carlo settings
☒	☐	For hierarchical and complex designs, identification of the appropriate level for tests and full reporting of outcomes
☐	☒	Estimates of effect sizes (e.g. Cohen's *d*, Pearson's *r*), indicating how they were calculated

Our web collection on statistics for biologists contains articles on many of the points above.

Software and code

Policy information about availability of computer code

Data collection	No software was used
Data analysis	The Methods text specifies code that has been posted to GitHub and is archived on Zenodo. All analyses were conducted using SAS version 9.4 (Cary, NC), R version 3.6.3 and 4.2.1 (Foundation for Statistical Computing, Vienna, Austria), or Epicure version 2.0 (Risk Sciences International, Ottawa, Canada).

For manuscripts utilizing custom algorithms or software that are central to the research but not yet described in published literature, software must be made available to editors and reviewers. We strongly encourage code deposition in a community repository (e.g. GitHub). See the Nature Portfolio guidelines for submitting code & software for further information.

Data

Policy information about availability of data

All manuscripts must include a data availability statement. This statement should provide the following information, where applicable:

- Accession codes, unique identifiers, or web links for publicly available datasets
- A description of any restrictions on data availability
- For clinical datasets or third party data, please ensure that the statement adheres to our policy

Raw molecular data are available from the Genomic Data Commons, accessed through the database of Genotypes and Phenotypes (dbGaP, accession phs001134; https://www.ncbi.nlm.nih.gov/gap/).

Research involving human participants, their data, or biological material

Policy information about studies with human participants or human data. See also policy information about sex, gender (identity/presentation), and sexual orientation and race, ethnicity and racism.

Reporting on sex and gender	The primary analyses included 335 females and 105 males. As specified in the Methods, regression analyses were adjusted for sex in addition to age at papillary thyroid cancer diagnosis.
Reporting on race, ethnicity, or other socially relevant groupings	The primary study population included all individuals from Ukraine. Analyses did not classify participants by race, ethnicity, or other socially relevant groupings.
Population characteristics	Study population characteristics are provided in Table S1.
Recruitment	The study population included a total of 359 individuals who were in utero or <=18 years of age on April 26, 1986 and lived in one of the regions of Ukraine most highly contaminated with 131I-containing fallout from the accident, and 81 individuals who were born >9 months after the accident and were therefore considered 131I-unexposed. All individuals had histologically-confirmed first primary papillary thyroid carcinoma with available biospecimens from the Chernobyl Tissue Bank and were included in a previous publication (Morton et al., Science 2021).
Ethics oversight	Informed consent for donation and research use was obtained from all participants or their legal representatives through the Chernobyl Tissue Bank (CTB). The study was approved by Institutional Review Boards at the patient treatment and tissue collection center (Institute of Endocrinology and Metabolism in Kyiv, Ukraine), the CTB coordination center (Imperial College of London, UK), and the United States National Cancer Institute.

Note that full information on the approval of the study protocol must also be provided in the manuscript.

Field-specific reporting

Please select the one below that is the best fit for your research. If you are not sure, read the appropriate sections before making your selection.

☒ Life sciences ☐ Behavioural & social sciences ☐ Ecological, evolutionary & environmental sciences

For a reference copy of the document with all sections, see nature.com/documents/nr-reporting-summary-flat.pdf

Life sciences study design

All studies must disclose on these points even when the disclosure is negative.

Sample size	All individuals with available samples were included, as described above.
Data exclusions	Exclusions were made based on poorly performing samples, as described in the Methods.
Replication	Findings were not replicated in an additional sample because all samples were utilized in the primary analysis.
Randomization	This was an observational study.
Blinding	All bioinformatics pipelines were run on all samples, without awareness of patient, clinical, or radiation exposure data.

Reporting for specific materials, systems and methods

We require information from authors about some types of materials, experimental systems and methods used in many studies. Here, indicate whether each material, system or method listed is relevant to your study. If you are not sure if a list item applies to your research, read the appropriate section before selecting a response.

Materials & experimental systems

n/a	Involved in the study	
☒	☐	Antibodies
☒	☐	Eukaryotic cell lines
☒	☐	Palaeontology and archaeology
☒	☐	Animals and other organisms
☒	☐	Clinical data
☒	☐	Dual use research of concern
☒	☐	Plants

Methods

n/a	Involved in the study	
☒	☐	ChIP-seq
☒	☐	Flow cytometry
☒	☐	MRI-based neuroimaging
